# Multimodality Imaging for Discordant Low-Gradient Aortic Stenosis: Assessing the Valve and the Myocardium

**DOI:** 10.3389/fcvm.2020.570689

**Published:** 2020-12-03

**Authors:** Ezequiel Guzzetti, Mohamed-Salah Annabi, Philippe Pibarot, Marie-Annick Clavel

**Affiliations:** Institut Universitaire de Cardiologie et de Pneumologie de Québec (Quebec Heart & Lung Institute), Quebec, QC, Canada

**Keywords:** aortic stenosis, low-gradient aortic stenosis, echocardiography, computed tomography, magnetic resonance imaging

## Abstract

Aortic stenosis (AS) is a disease of the valve and the myocardium. A correct assessment of the valve disease severity is key to define the need for aortic valve replacement (AVR), but a better understanding of the myocardial consequences of the increased afterload is paramount to optimize the timing of the intervention. Transthoracic echocardiography remains the cornerstone of AS assessment, as it is universally available, and it allows a comprehensive structural and hemodynamic evaluation of both the aortic valve and the rest of the heart. However, it may not be sufficient as a significant proportion of patients with severe AS presents with discordant grading (i.e., an AVA ≤ 1 cm^2^ and a mean gradient <40 mmHg) which raises uncertainty about the true severity of AS and the need for AVR. Several imaging modalities (transesophageal or stress echocardiography, computed tomography, cardiovascular magnetic resonance, positron emission tomography) exist that allow a detailed assessment of the stenotic aortic valve and the myocardial remodeling response. This review aims to provide an updated overview of these multimodality imaging techniques and seeks to highlight a practical approach to help clinical decision making in the challenging group of patients with discordant low-gradient AS.

## Introduction

Discordant low-gradient aortic stenosis (AS) (i.e., an aortic valve area ([AVA] ≤ 1 cm^2^ with a mean transvalvular gradient <40 mmHg and/or peak jet aortic velocity <4 m/s) is a frequent finding, with up to 40% AS patients harboring discrepant results at transthoracic Doppler-echocardiography (TTE) examination (1). From a fluid dynamics standpoint, for a normal mean flow rate of 200–250 ml/s the value of AVA that corresponds to a MG of 40 mmHg is 0.8 ± 0.1 cm^2^ rather 1.0 cm^2^ (2). The current paradigm that considers AS a disease of the aortic valve but also of the left ventricular (LV) myocardium (3, 4) is especially true for discordant low-gradient AS, and particularly for those with low-flow (i.e., a stroke volume index [SVi] ≤ 35 ml/m^2^). Indeed, the discordant grading pattern raises challenges and uncertainties regarding the true severity of the valve disease, while low-flow, which is the common endpoint of the particularly deleterious combination of increased afterload and decreased myocardial performance (e.g., decreased contractility, adverse LV remodeling, etc.) (5, 6) increases risk of adverse outcome.

Therefore, a comprehensive approach must be adopted to: (i) confirm the actual severity of the valve disease; and (ii) assess the degree of myocardial damage caused by (or concomitant with) AS.

## Classification of Low Gradient AS

**Figure 1:**
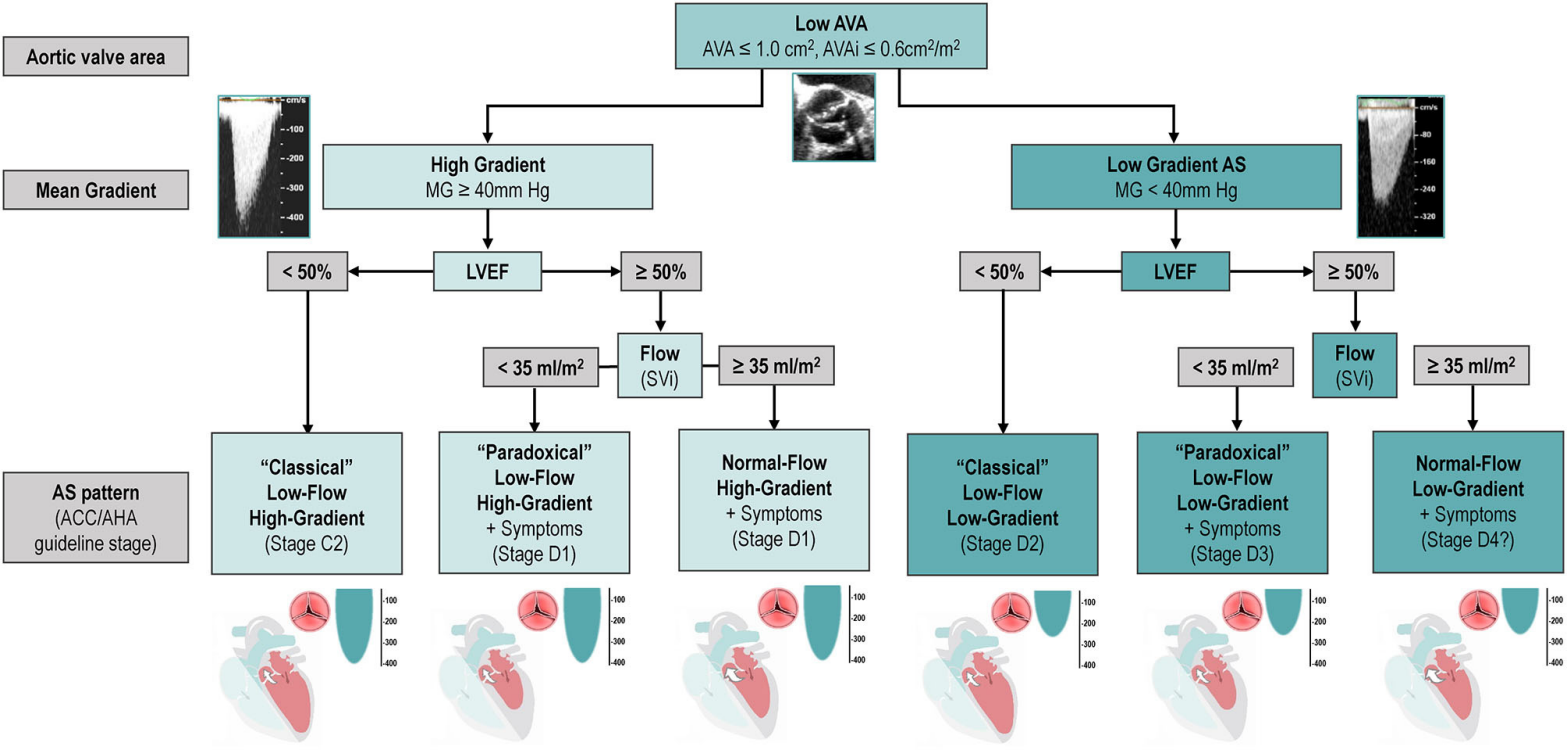
Classification and Characterization of the different types of AS according to AVA, Gradient, LVEF and Flow. The classification of types of AS, including only the categories associated with symptoms and/or depressed LVEF. It does not include stage C1 (i.e., patients with high-gradient AS, no symptoms, and preserved LVEF). Question mark indicates stage labels that are proposed by the authors but are not included in the guidelines and will need to be further tested and validated. ACC, American College of Cardiology; AHA, American Heart Association; AS, aortic stenosis; AVA, aortic valve area; AVAi, indexed aortic valve area; LVEF, left ventricular ejection fraction; MG, mean gradient; SVi, stroke volume index.

In patients with small AVA (≤ 1 cm^2^) and low gradient (<40 mmHg), several subtypes can be defined according to the left ventricular ejection fraction (LVEF) and flow status (Figure 1).

### “Classical” (Reduced LVEF) Low-Gradient AS

“Classical” (Reduced LVEF) low-gradient AS defined in guidelines as the combination of LVEF <50%, AVA ≤ 1 cm^2^ and a mean gradient <40 mmHg (7, 8). Found in 5–10% of AS population (1), it is associated with worse outcomes under both clinical management and after aortic valve replacement (AVR). Although the decreased LVEF might be theoretically caused exclusively by a very severe AS (i.e., afterload mismatch), it is generally due to a combination of high afterload due to valvular disease and intrinsic myocardial impairment, due to ischemic heart disease, diffuse/focal myocardial fibrosis secondary to AS/hypertension or concomitant cardiomyopathies (9, 10). Although most patients with classical low-gradient AS typically have a low flow state, some may nonetheless have a normal SVi.

### “Paradoxical” (Preserved LVEF) Low-Flow Low-Gradient AS

“Paradoxical” (Preserved LVEF) low-flow low-gradient AS defined as an AVA ≤ 1 cm^2^, a mean gradient <40 mmHg, a SVi ≤ 35 ml/m^2^ and an LVEF ≥50% (7, 8). Due to the fact that mean gradient is more dependent on transvalvular volumetric flow rate [FR] (understood as the volume of fluid which passes per unit time and calculated as the stroke volume divided by the left ventricle ejection time) than on SVi, some investigators propose to define low-flow as a mean FR <200 ml/s (11, 12). Furthermore, recently published sex-specific cut-points (<32 ml/m^2^ in women and <40 ml/m^2^ in men) seemed to improve the prediction of outcomes after surgical AVR (13). Low SVi and/or FR despite normal LVEF can be linked to various factors such as small LV cavities (particularly in older hypertensive women with small body size), low GLS, severe diastolic dysfunction especially restrictive filling physiology or pericardial constriction, atrial fibrillation, significant mitral regurgitation or stenosis, pulmonary hypertension, and tricuspid regurgitation (14, 15).

**Figure 2:**
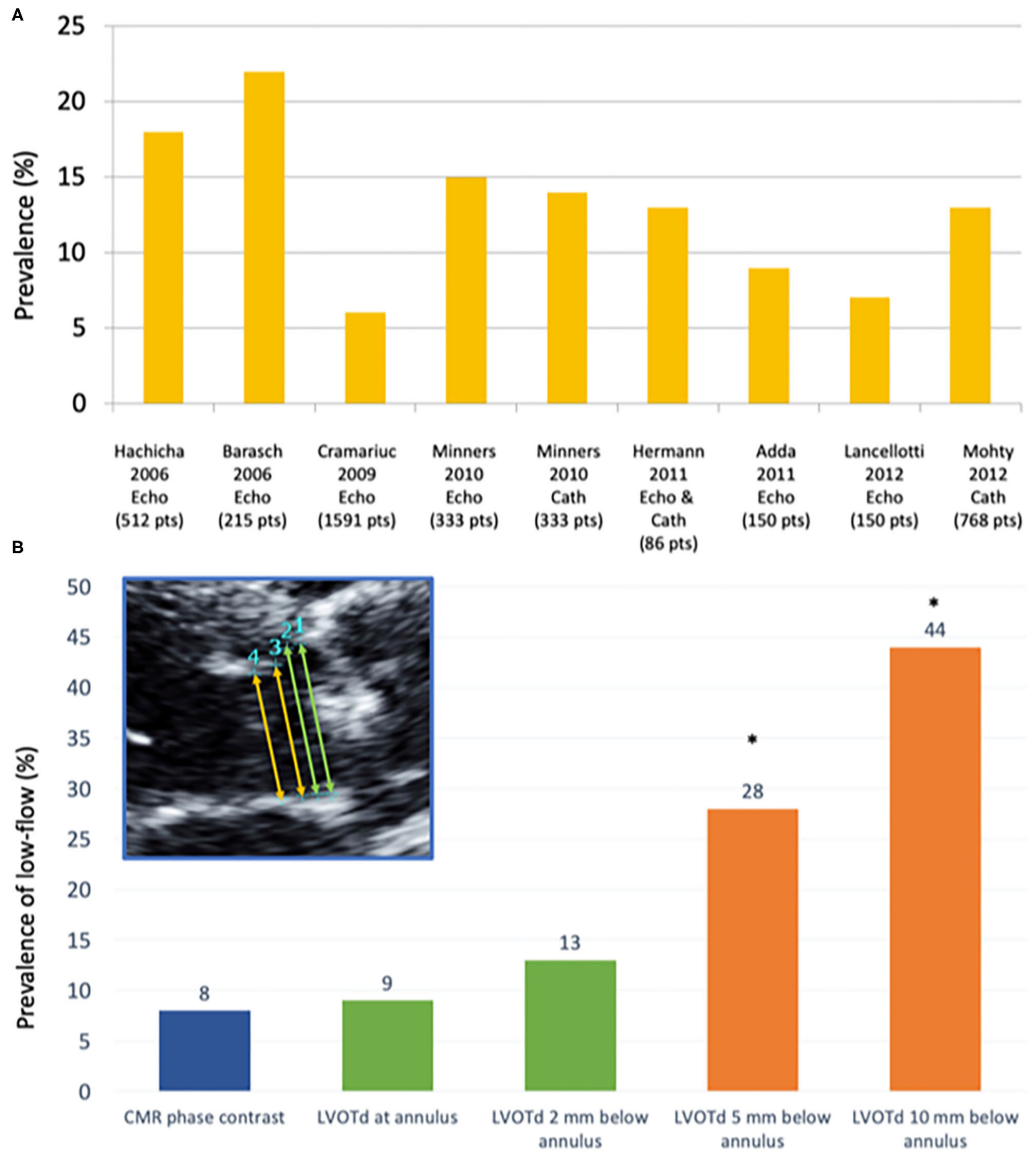
Prevalence of low flow according to different studies and measurement techniques. **(A)** Prevalence of paradoxical low-flow severe AS (i.e., AVA <1.0 cm^2;^ MG <40 mmHg, LVEF>50%; SVi <35 mL/m^2^) according to different studies using echocardiography (Echo) and/or invasive catheterization (Cath). **(B)** Prevalence of low-flow state (stroke volume index ≤ 35 ml/m^2^) according to different measurement sites of the LVOT: (1) at the hinge points of the aortic valve leaflets (annular level); (2) very close to (i.e., 2 mm below) the annular level; (3) 5 mm below the annular level; and (4) 10 mm below the annular level, as compared to the referent standard (phase-contrast CMR) [modified from JASE (16)]. CMR, Cardiovascular magnetic resonance; LVOTd, left ventricular outflow tract diameter. *p < 0.01 as compared to CMR-PC (referent method).

Prevalence of low-flow low-gradient AS and preserved LVEF shows high variability (i.e., between 5 and 35%) according to the institution/country (Figure 2A). Patients with this entity are more frequently women, with pronounced concentric remodeling and small LV cavity, diastolic dysfunction and reduced LV systolic longitudinal function despite the preserved LVEF (15). Finally, up to one third paradoxical low-flow low-gradient patients might have concomitant cardiac amyloidosis (17), which is usually associated with decreased longitudinal function albeit an LVEF ≥50%.

### Normal-Flow Low-Gradient AS

Normal-flow low-gradient AS defined as an AVA ≤ 1 cm^2^, AVAi ≤ 0.6 cm^2^/m^2^, LVEF ≥50% and a SVi >35 ml/m^2^, it has only recently been incorporated into guidelines (7). ESC/EACTS guidelines advocate the idea that this entity corresponds to a moderate stage of the disease and therefore a conservative approach is warranted. There is strong evidence, however, that a significant proportion of normal-flow low-gradient AS patients have truly severe AS and would benefit from AVR (18–20). From a pathophysiological and fluid dynamics standpoint, the presence of a low-gradient despite a normal SVi is explained by several factors, even after excluding measurement errors and the inherent inconsistencies of guidelines criteria (18, 21): the first and more obvious is the presence of an actually decreased FR despite a normal SVi, such as the case of bradycardia and a prolonged ejection time (18). The other main factor affecting the AVA-gradient relationship and leading to a normal-flow low-gradient pattern is due to abnormal arterial hemodynamics: the presence of systemic hypertension and/or reduced arterial compliance has been shown to decrease SVi, prolong left ventricular ejection time and cause a drop in mean transvalvular flow rate and/or mean gradient (18, 22).

## Multimodality Imaging In Low-Gradient AS

Due to the limited sensitivity and specificity of physical examination in the diagnosis of valve diseases (23), non-invasive cardiac imaging has become paramount in their evaluation (4, 24). Imaging can assess two of the three triggers for intervention (i.e., stenosis severity and myocardial damage). Due to its wide availability, relatively low-cost and high performance, TTE is the first method of choice and the cornerstone of AS evaluation: it can assess the valve severity and its consequences on the LV myocardium, such as LV hypertrophy, remodeling and ejection fraction. However, other imaging modalities such as multidetector computed tomography (MDCT) and cardiovascular magnetic resonance (CMR) have recently emerged as invaluable tools for the assessment of both AS severity (7, 25–27) and myocardial damage (3, 28, 29). Furthermore, non-invasive cardiac imaging (especially MDCT) has a central role in planning and follow-up of transcatheter AVR (30). Finally, positron-emission tomography has recently been shown to be an early marker of valve inflammation and calcification (31, 32), as well as of subclinical bioprosthetic valve deterioration (33).

## Assessment of Valve Disease Severity

The first step when facing a patient with low-gradient AS is to confirm the actual severity of AS. Assessment of AS severity is mostly based on three parameters (Table 1): peak transaortic jet velocity (Vmax), mean pressure gradient and AVA (i.e., effective orifice area) (7, 8, 37). However, by definition low-gradient patients will have a MG <40 mmHg and in a vast majority of cases a Vmax <4 m/s, as both parameters are highly dependent on transvalvular flow (9, 38) and highly correlated (*r*^2^ = 0.98) (2).

**Table 1:** Imaging markers to assess severity of valvular damage in aortic stenosis.

**Imaging modality**	**Cut-off for severe**	**Concept**	**Advantages**	**Limitations**
**Rest echocardiography**				
AS jet velocity	>4.0 m/s	Velocity increases as AS severity increases	Direct measurement Extensive evidence	Flow-dependent Misalignment frequent
Mean gradient	>40 mmHg	Pressure gradient calculated from velocity (simplified Bernoulli equation)	More reproducible than peak velocity Extensive evidence	Flow-dependent Misalignment frequent Pressure recovery phenomenon
Aortic valve area (Effective orifice area)	<1.0 cm^2^	Flow proximal to the valve and in the EOA is equal *(continuity equation)*	Measures the hemodynamic EOA Extensive evidence Less (but still) flow-dependent than peak velocity and mean gradient	Requires multiple measurements (AV and LVOT velocities and LVOT area). LVOT area particularly subject to measurement errors/geometric assumptions
Aortic valve area index	<0.6 cm^2^/m^2^	EOA adjusted for body surface area	Increase specificity in low BSA (especially women); increase sensitivity in high BSA (especially men)	Not valid for obese (BMI >30 kg/m^2^) Less extensive evidence than for AVA 1.0 cm^2^ Still flow-dependent
Velocity ratio	<0.25	EOA expressed as a proportion of LVOT area	Doppler-only method No need for LVOT diameter measurement and no geometric assumptions	Limited evidence No information on stroke volume Still flow-dependent
Aortic valve area (Planimetry)	<1.0 cm^2^	Anatomic (geometric) AVA	No Doppler information needed	GOA ≥ EOA (flow contraction) Still flow-dependent (in low-flow status AV opening is restricted) Technically challenging (echogenicity/calcification) Low reproducibility
Energy loss index	<0.5 cm^2^/m^2^	EOA corrected for distal recovered pressure in ascending aorta	Theoretically closer to true hemodynamic burden caused by AS Relevant in patients with small aorta (<3 cm)	More complex and prone to errors Most patients with AS have aortic diameters >3 cm Still flow-dependent
**Dobutamine stress echocardiography**				
Projected AVA at normal flow rate (34)	<1 cm^2^ (<1.2 cm^2^)	Estimation of AVA at normal flow rate (250 ml/s) by plotting AVA vs. flow and calculating the slope	Accounts for variable changes in flow during low-dose dobutamine stress echocardiography. Improved outcome prediction and true severity of AS prediction than conventional DSE parameters	Requires DSE (small but non-negligible risk) Requires ≥15% increase in mean flow rate Expertise required and time-consuming
**Multidetector computed tomography**				
AV calcium score (non-contrast) (25, 35, 36)	♀≥1200 AU ♂≥2000 AU	Semi-quantitative (Agatston method) assessment of AV calcification (anatomical severity)	Excellent correlation with hemodynamic (AVA and MG) and anatomical (valve weight) severity 100% flow-independent Low radiation (<1 mSv) Good predictor of clinical outcomes Simple and reproducible	Availability of CT required Unable to measure fibrosis May underestimate severity in young bicuspid?, Asian ethnicity? Variability in extremely small/large annuli No hemodynamic information
AV calcium score density (25, 35, 36)	♀≥292 (≥420) AU/cm^2^ ♂≥476 (≥527) AU/cm^2^	AV Calcium Score adjusted for LVOT area (echo)		Same as AV calcium score Prone to errors and decreased reproducibility as it incorporates LVOT diameter by echocardiography
**Cardiovascular magnetic resonance**				
AS jet velocity	>4.0 m/s	Same as above	No angle-interrogation or echogenicity limitations Optimal off-line alignment (4D flow acquisitions)	Systematic underestimation of peak velocities vs. echo (insufficient spatiotemporal resolution/partial volume effect)
Aortic valve area (Planimetry)	<1.0 cm^2^	Same as above	No acoustic window limitations	Same as above Poor visualization of calcium in CMR Partial volume effect

*Adapted from (37). AU, Agatston Units; AV, aortic valve; AVA, aortic valve area; CMR, cardiovascular magnetic resonance; EOA, effective orifice area; GOA, geometric orifice area; LVOT, left ventricular outflow tract; DSE, dobutamine stress echocardiography*.

### Validation of Rest Echocardiographic Parameters

**Figure 3:**
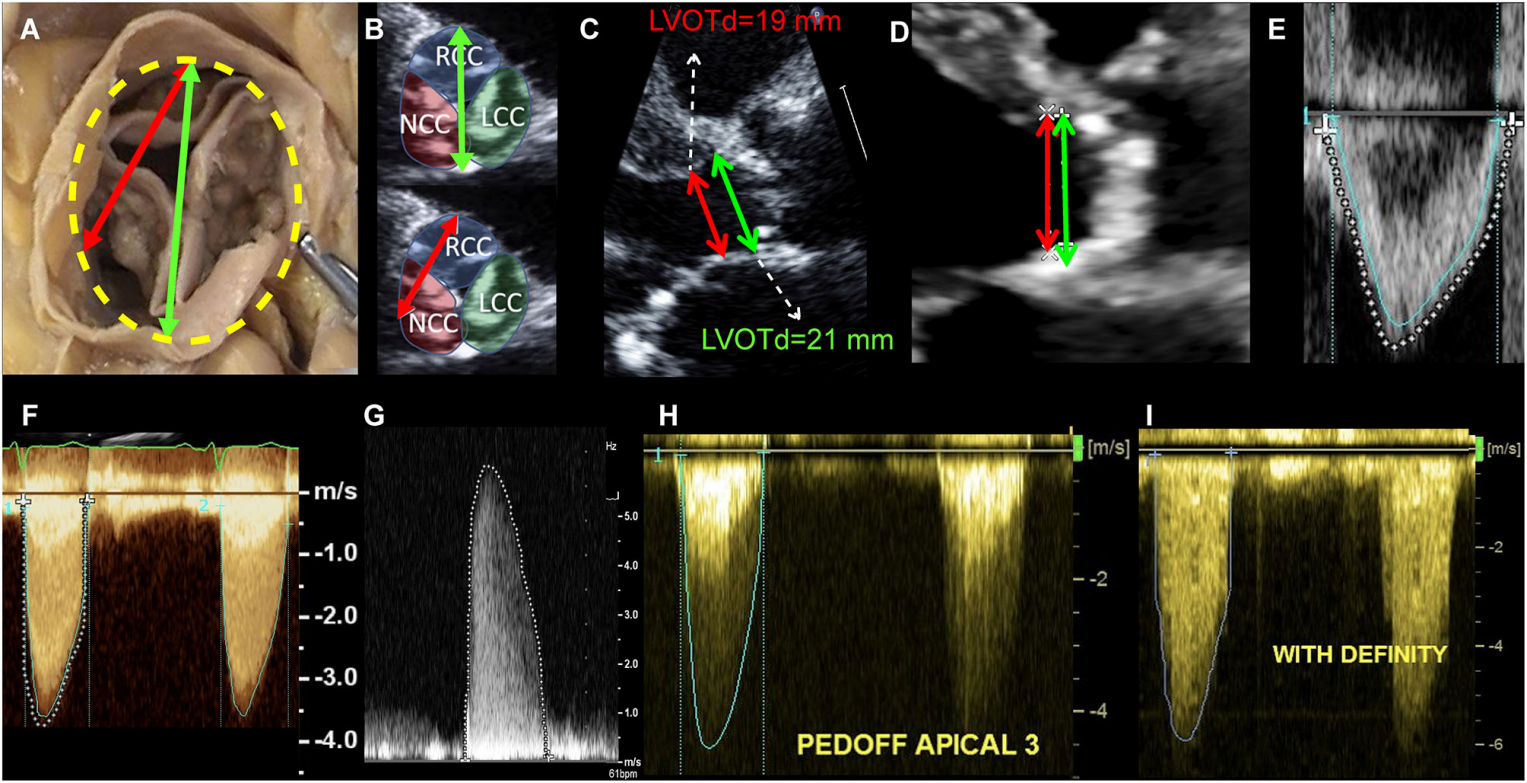
Echocardiographic measurement pitfalls and how to avoid them. LVOT diameter must be measured at its maximal dimension, which generally corresponds to the bisection between the right coronary cusp hinge point anteriorly and the interleaflet triangle between the left and non-coronary cusps posteriorly **(A,B)**. LVOTd must be measured at the annulus and not 5–10 mm below, as this leads to significant underestimation of AVA and SV **(C)**. In this case, measuring 5 mm below the annulus (as recommended in guidelines) lead to an LVOT area of 2.83 cm^2^, as compared to an area of 3.46 cm^2^ when measured at the annulus (18% underestimation, therefore leading to significant underestimation of SV and AVA). In case of LVOT ectopic calcification, if the plane that bisects the largest diameter cannot exclude the calcium, LVOT diameter measurement should include the calcium in the measurement **(D)**. LVOT velocity-time integral should be measured at the modal velocity (the densest line of the Pulse Wave Doppler) since flow at the LVOT is laminar (**E**: blue trace represents the modal velocity whereas the dashed white line overestimates LVOT VTI). For an accurate measurement of the transaortic jet velocity, tracing should be done at peak velocities but excluding fine linear signals (**F**: green trace represents the correct measurement, whereas the dashed white line overestimates aortic valve VTI by including linear signals). It is paramount that the Doppler beam is optimally aligned parallel to the stenotic aortic jet. Therefore, a meticulous search of the highest transvalvular velocity is mandatory. This requires a comprehensive Doppler study that is not only limited to the apical window but also includes right parasternal, suprasternal, and sometimes subcostal approaches using a small, dedicated CW Doppler transducer (*pencil probe* or *Pedoff transducer*) **(G,H)**. Finally, the use of ultrasound enhancing agents (i.e., contrast echocardiography as with Definity®) might lead to overestimation of transvalvular velocities and gradients and therefore caution should be taken **(I)**.

The first and most important aspect is to effectively ensure that a low gradient and a low velocity are present. The importance of a careful and comprehensive multi-window acquisition cannot be emphasized enough, since in up to 50% of patients with AS the peak velocity is obtained in a right parasternal view (37, 39, 40). However, a recent EACVI survey including 125 centers from 32 countries (87% of them tertiary/university high-volume centers) showed that almost half of them did not routinely obtained measurements from the right parasternal view (41). Therefore, before moving to advanced non-invasive imaging, it is fundamental to ensure an optimal echocardiographic acquisition (Figure 3). The choice of the appropriate view can be guided by the jet direction, but we suggest to systematically acquire all windows (apical 5 and 3 chamber, right parasternal, subcostal) in order to ensure optimal alignment with the jet and adequate recording of the true (highest) gradients. Otherwise, an apparently “low-gradient” AS might just be an incompletely evaluated true high gradient AS.

The effective AVA is calculated based on the conservation of mass principle that the SV ejected through the left ventricular outflow tract (LVOT) all passes through the stenotic orifice; thus, SV at the valve is equal to the SV at the LVOT (i.e., continuity equation). The LVOT is assumed to be circular and its area calculated as:

$$\begin{array}{l} LVOTarea\;=\pi *{\left(\frac{\text{LVOTdiameter }}{2}\right)}^{2} \end{array}$$

AVA is then calculated as follows:

$$\begin{array}{l} AVA\;=LVOTarea*\left(\frac{\text{LVOT VTI }}{\text{Aortic VTI }}\right) \end{array}$$

where LVOT VTI and Aortic VTI are the velocity-time integrals of the LVOT and aortic flow, respectively. Importantly, due to the flow contraction phenomenon at the jet's narrowest part (vena contracta), the effective AVA is ~10–20% smaller than the actual orifice area i.e. geometric AVA, from which it should be distinguished (42).

The greatest potential of error is the measurement of LVOT diameter; given that it must be squared, a small error in LVOT diameter may result in important errors in the calculation of SV and, thus, AVA (43). Furthermore, studies using 3 dimensional (3D) methods such as cardiovascular magnetic resonance (CMR), computed tomography (CT) and 3D echocardiography have shown that LVOT is indeed not circular but elliptic (27, 37, 44–46), and that this ellipticity is more pronounced in the region farther from the annular plane (45). The fact that the LVOT is indeed not circular and that two dimensional (2D) TTE measures its minor-axis is one of the main causes of underestimation of LVOT area and, potentially, SV and AVA (47–49). We recently showed that the site of LVOT diameter measurement [i.e., at the annulus or 5-10 mm below as recommended by guidelines (37)] has a major impact on the estimation of AVA and SV and, therefore, on the prevalence of severe AS and low-flow (16) (Figure 2B). Furthermore, measurement at the annulus is more reproducible and shows best agreement with non-invasive (16), invasive methods (50) and has an equivalent predictive value that 3D methods (46). A simple, comprehensive checklist on LVOT measurement by TTE has been previously described (43).

Other proposed TTE parameters that may help are (Table 1):

- Velocity ratio: Also known as Doppler velocity index or dimensionless index, it is the ratio of Vmax or VTI between the LVOT and the aortic valve (i.e., vena contracta) (51). Normal value is close to 1, whereas a severe AS is present when ratio is <0.25. This is a helpful parameter when LVOT diameter is doubtful or cannot be measured and it has been proven to be associated to worse outcomes (52). However, despite being less flow than gradient/velocity, DVI remains a flow-dependent parameter (51).
- Acceleration time (AT): An echocardiographic representation of the classical semiological finding of *pulsus parvus et tardus* of severe AS. AT is defined as the delay between the beginning and the peak aortic ejection velocity and the ejection time (ET) is the total ejection time. An AT >94 ms and an AT/ET >0.35 (or 0.36) have been proposed as parameters with good sensitivity and specificity to identify severe AS (53–55). The main limitation of these studies is the lack of a flow-independent gold standard for assessment of severe AS. Furthermore, they should be used with caution in patients with low-flow AS, since ejection dynamics are altered and validity of these parameters have not been proven in this population.
- AVA planimetry (geometric AVA): It refers to the measurement of the geometric AVA by direct anatomic visualization. However, both theoretical and practical important caveats exist. From a fluid dynamic standpoint, the ratio of effective orifice area/geometrical orifice area (i.e. contraction coefficient) varies from 0.61 to 1 according to shape of the valve (56), the flow rate (57), etc. Thus, though the same cut-off is generally used (<1 cm^2^) the geometrical orifice area is larger than the effective orifice area (42) and for an effective AVA of 1 cm^2^, the geometric AVA could varies from 1 to 1.6 cm^2^. Nevertheless, in practice, a proper alignment at the tip of valve leaflets must be ensured in order to avoid overestimation, and therefore it should be performed using a 3D imaging modality (ideally 3D-transesophageal echocardiography). Planimetry is difficult when extensive calcifications with acoustic shadows and reverberations are present. Furthermore, planimetric AVA is less reproducible and lacks outcome data as compared to other more established methods (46, 58). Finally, in cases of low-flow and/or low gradient, in which valve-opening forces are reduced, a pseudo-severe AS cannot be ruled out on this parameter alone. These limitations, along with its invasive nature, precludes TEE-planimetry to become the first choice to confirm the true severity of AS. 3D-TEE is, however, an excellent alternative to MDCT for pre-procedural evaluation for TAVR and it is extremely useful for assessment of concomitant valve disease and, in some patients, a deep transgastric view can be useful to obtain peak aortic velocity/gradients. MDCT and/or CMR can be used as well for AVA planimetry, but some of the aforementioned limitations persist.
- Energy loss index: The fluid energy loss across a stenotic valve is affected by not only by the valve effective orifice area but also by flow rate and downstream aortic cross-sectional area (Aa), which can significantly affect the amount of kinetic energy that is transformed into hydraulic pressure, a phenomenon known as *pressure recovery* (59). Pressure recovery is more pronounced in cases with large EOA/Aa ratios and can therefore be clinically meaningful in patients with small aortas. To account for this pressure recovery phenomenon, the concept of energy loss index (ELI) was proposed (60): ELI = AVA × Aa/(Aa–AVA)/BSA, where Aa is the aortic area at the level of the sinotubular junction and BSA is the body surface area. This parameter, which takes into account the ratio between the EOA and the aortic area (Aa), provides an accurate estimation of the energy loss due to AS (mostly secondary to flow separation distal to the stenosis) and has been proven to provide independent and incremental prognostic information in addition to standard measures of AS severity (61, 62). Its application, however, was never generalized due its added complexity and the fact that most patients with AS present with an Aa diameter >3 cm.

Briefly, in case of discordant grading (MG <40 mmHg and Vpeak <4 m/s and AVA ≤ 1 cm^2^ and AVAi ≤ 0.6 cm^2^/m^2^), before moving forward to more advanced methods, one must ensure that TTE measurements were properly acquired: 1) Ensure patient is not hypertensive (SBP ≥160 mmHg) at the moment of the exam, as it may decrease transvalvular gradients; 2) Ensure multi-window interrogation (including right-sternal border), ideally with a dedicated dual-crystal Pedoff transducer; 3) Make sure LVOT was appropriately measured at the annulus level (not 5-10 mm below) in a mid-systolic frame that bisects the largest dimension (i.e., the plane that bisects the right coronary cusp hinge point anteriorly and the interleaflet triangle between the left and non-coronary cusps posteriorly) (Figure 3). Care should be taken in case of extensive calcifications (which should be included in the diameter measurement). If necessary, calculate predicted LVOT diameter using the formula: *LVOTd* = (5.7 *x BSA*)+12.1 (in mm) and, if measured LVOT is ≥2 mm smaller or larger, suspect error in measurement. Confirm that velocity ratio ≤ 0.25 and/or ELI ≤ 0.6cm^2^/m^2^; 4) Corroborate calculated SV with that obtained with 3D-echo (if available) or biplane Simpson method [which, if appropriately done−ensuring no foreshortening and appropriate tracing of endocardial borders at the compact portion of the myocardium excluding trabeculations−shows excellent agreement with Doppler-methods and PC-CMR (16)].

### Stress Echocardiographic Evaluation

Low-dose dobutamine stress echocardiography (DSE) has been used to differentiate pseudo-severe AS [which *a priori* does not benefit from AVR, though there is an ongoing trial evaluating the utility of TAVR in moderate AS with depressed LVEF (63)] from true severe AS (which is expected to benefit from AVR). The protocol for stress echocardiography for evaluation of LF-LG AS begins with a dose of 5 μg/kg/min with incremental increases every 3–5 min to a maximum dose of 20 μg/kg/min. The rationale is to generate an increase in stroke volume (due to an increase in contractility) and/or an increase in mean flow rate (combined increase in contractility and reduction of ejection time) to provoke changes in mean gradient and valve area. Traditionally, the presence of flow reserve (i.e., an increase in SV ≥20%) has been considered a marker of better postoperative prognosis and a condition for proper assessment of the true severity of AS. In patients with flow reserve, an increase in MG to ≥40 mmHg and/or Vpeak to ≥4 m/s with an AVA that remained <1.0 cm^2^ is considered to have true-severe AS, whereas a patient in whom AVA increased to ≥1.0 cm^2^ and MG remains below 40 mmHg (Vpeak below 4 m/s) is considered to have moderate AS (7, 8). However, a significant proportion of patients do not have FR [8 to 30% according to the parameter used (64, 65)] and/or have inconclusive results at DSE [up to 50% (34)]. Hence, the concept of projected AVA (i.e., the projected AVA at a normal flow rate of 250 ml/s) was developed (34, 64, 66). After an increase of ≥15% in flow rate, using AVA and flow rate [Q], projected AVA is calculated as follows:

$$\begin{array}{l} AVA_{Proj}=AVA_{Rest}+\frac{AVA_{Peak}-AVA_{Rest}}{Q_{Peak}-Q_{Rest}}\;x\;\left(250-Q_{Rest}\right) \end{array}$$

A projected AVA ≤ 1 cm^2^ (or projected AVA index ≤ 0.55 cm^2^/m^2^) is considered severe. In many ways the concept of projected AVA outperforms the traditional flow-reserve concept, which may be intrinsically flawed due to the complex interaction between decreased contractility, increased afterload and altered geometry in classical low-flow AS (67). Furthermore, a recent study showed that myocardial fibrosis (focal and diffuse) was no different in patients with and without FR (10), further challenging the concept that absence of FR is a valid surrogate marker of intrinsic myocardial impairment. DSE may also be used to confirm the presence of severe AS in patients with paradoxical low-gradient AS (if Q at rest <250 ml/s) (68). However, many patients with paradoxical low-flow low-gradient AS have small ventricles, with concentric remodeling and a high prevalence of atrial fibrillation and isolated upper septal hypertrophy, which may lead to serious side effects (LVOT dynamic obstruction with hypotension, syncope, etc.). Thus, DSE if needed, should be performed with caution and stopped as soon as increase in flow is sufficient, which is often before 20 μg/kg/min) (68). In asymptomatic patients with paradoxical low-flow low-gradient AS, the use of exercise echocardiography may serve to simultaneously assess symptomatic status and severity of AS with the projected AVA (a “two birds, one stone” approach) (68). Nevertheless, an easier and faster alternative approach (i.e., aortic valve calcium [AVC] quantification by MDCT) is often preferred.

### Multidetector Computed Tomography in AS

**Figure 4:**
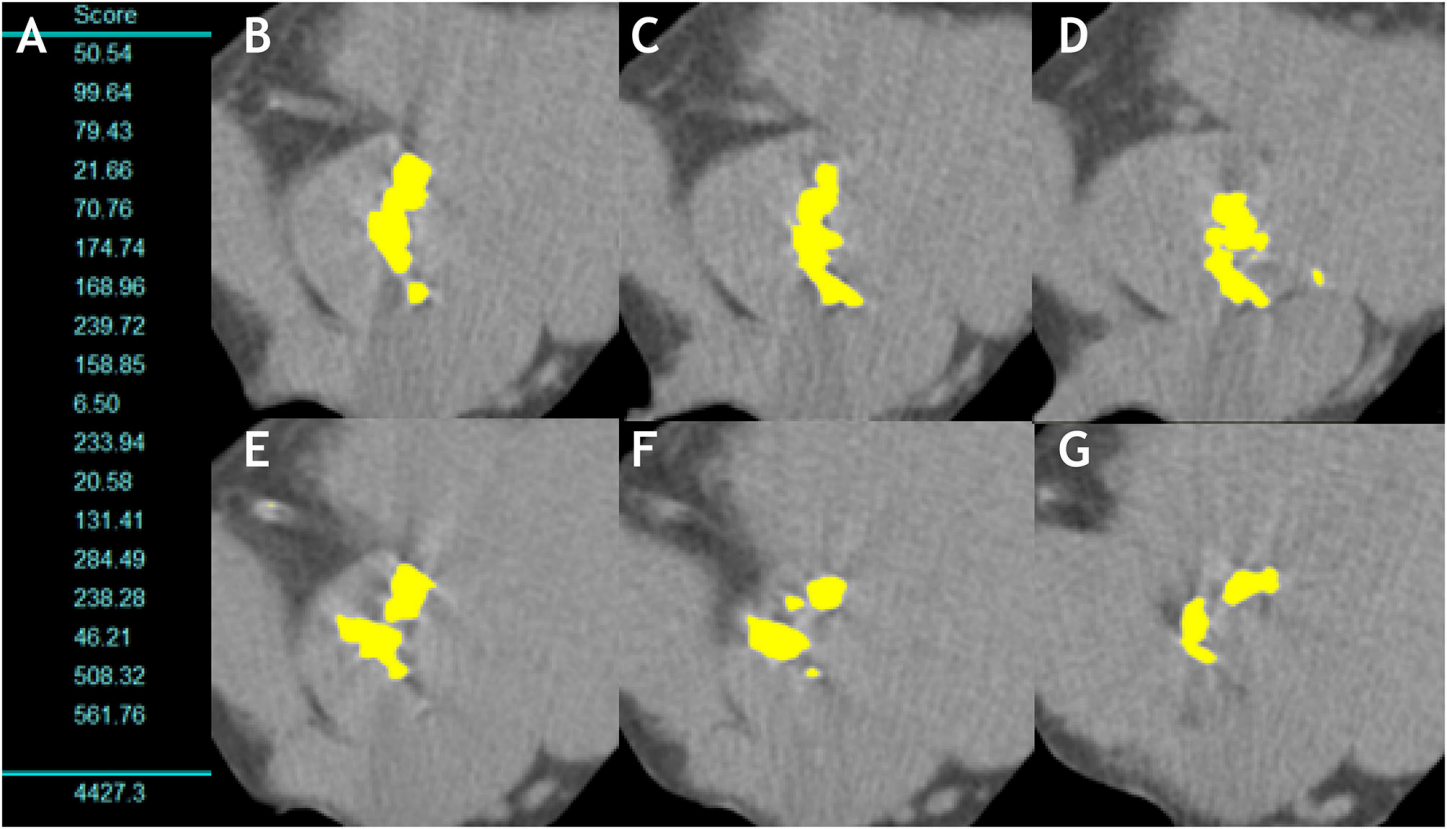
Aortic valve calcification measurement in a man with severe aortic stenosis. **(A)** Shows the measurement of the aortic valve calcium in each 3-mm slice and total sum (AVC score = 4,427.3AU). **(B–G)** Show the multiple axial images from aortic annulus to aortic root with any aortic valve calcification highlighted in yellow by the software.

The advent of AVC quantification using MDCT has revolutionized AS evaluation in the last years (Figure 4). It requires an ECG-gated, non-contrast acquisition with low radiation (<1 mSv). Quantification relies on the Agatston method using semi-automated software and provides a flow-independent quantitative assessment of AS anatomical severity. Details on the scan protocol, quantitative analysis and caveats have been recently published (35). It is especially important to avoid including coronary, LVOT and mitral valve calcium into the quantification of AS severity. Though multiplanar reconstruction is important for anatomical orientation and careful exclusion of these structures, measurements should be done in the axial stacks and not in the *en face* reconstructed sequences, since this leads to significant underestimation of AVC score (69). Considering the appropriate technique is used, AVC quantification has excellent reproducibility and performance for AS severity assessment and has consistently proven to be of incremental prognostic value in several studies (25, 36). Furthermore, it has been recently incorporated into ESC/EACTS guidelines to be used in patients with symptomatic, discordant grading AS and both reduced (<50%) and preserved LVEF (≥50%) (7). Due to sex-differences in AS pathophysiology [women present with more fibrosis and less calcification than men (70)], specific cut-offs defining severe AS differ for women (≥1,200 Agatston Units [AU]) and men (≥2,000 AU). These cut-points have been validated in mostly Caucasian cohorts (25, 36) and have shown a sensitivity of 80–89% and a specificity of 81–84% (25, 26, 35).

However, some studies suggested that ethnic differences may exist in valvular calcification (71, 72). We recently analyzed an international multicentric registry (to be published) including 1,263 patients (57% Asian, 45% women) and showed that accuracy of guidelines' cut-point (≥2,000 AU) was high and comparable among Caucasian and Asian men (88 and 84% respectively, *p* = 0.21), whereas the ≥1,200 AU cut-point provided significantly lower correct classification of AS for Asian women as compared to Caucasian women (76 vs. 94% respectively, *p* < 0.001) (73). Accuracy of AVC density (total AVC divided by LVOT area calculated by transthoracic echocardiography, with cut-points of 476 AU/cm^2^ in men and 292 AU/cm^2^ in women) was comparable to absolute AVC in Caucasians (91 vs. 91% respectively, *p* = 0.74), but higher than absolute AVC in Asians (87 vs. 81%, *p* < 0.001).

Regarding bicuspid morphology, we analyzed 485 patients with bicuspid aortic valves and found that previously defined AVC (2065 and 1274 AU in men and women, respectively) and AVC density (476 and 292 AU/cm^2^) had sensitivities of 80–83% and a specificity of 82% for defining severe AS (to be published).

Therefore, calculation of AVC density provides an important step in specific challenging cases (i.e., bicuspid morphology, non-caucasian women) and seems to better predict outcomes under medical management (36).

**Figure 5:**
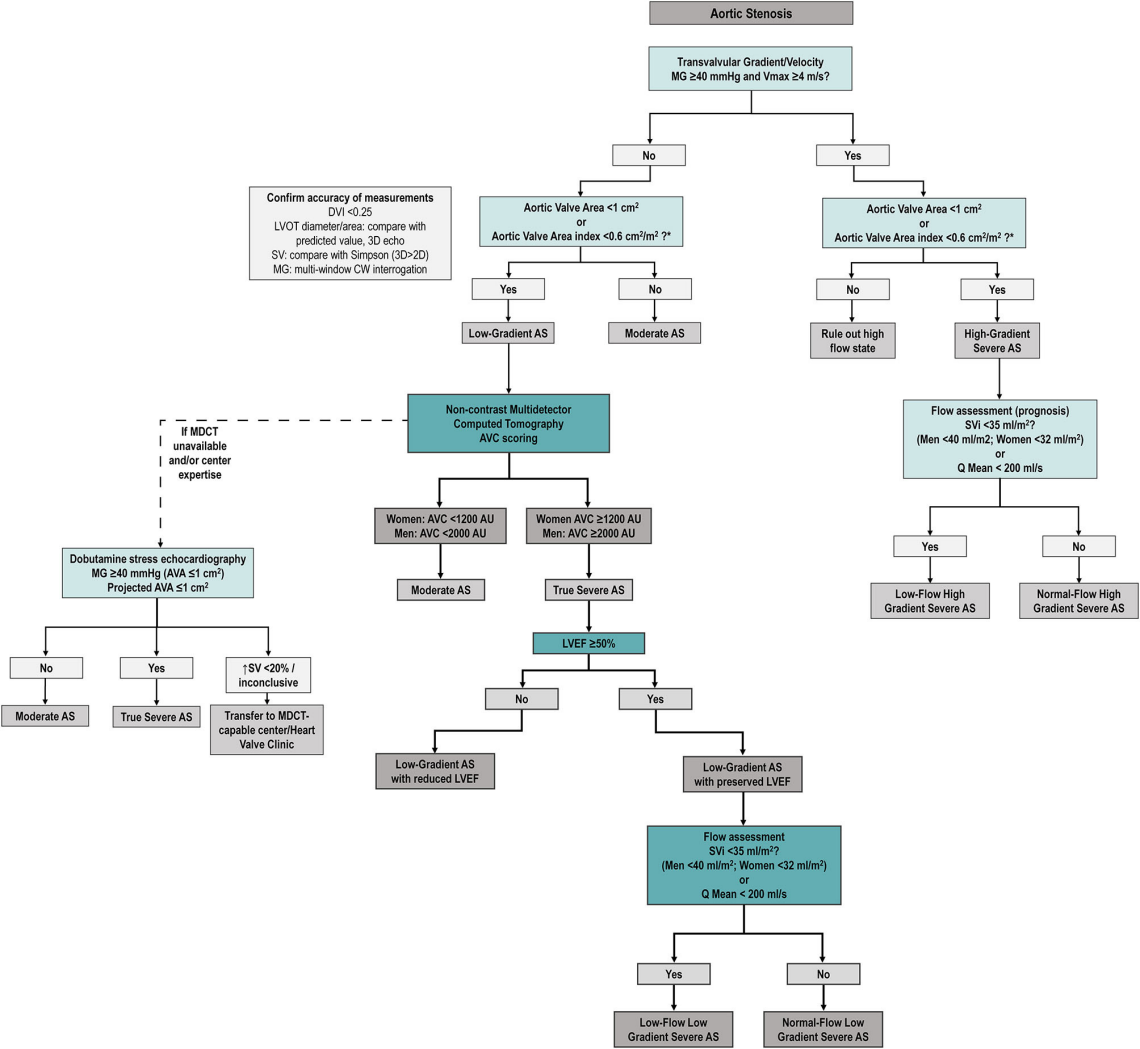
Proposed diagnostic algorithm for severe aortic stenosis. Proposed algorithm for diagnostic assessment of AS. As compared to ESC/EACTS guideline algorithm (7), we suggest assessing SVi (and/or Q mean) in all AS patients (regardless of mean gradient). After careful revision of accuracy of echocardiographic measurements (left box), MDCT AVC is proposed to confirm true anatomical severity in all discordant grading patients (i.e., low AVA and low gradient/Vmax) regardless of LVEF and SVi. Dobutamine stress echocardiography remains as an alternative option if MDCT unavailable (see text). AS, aortic stenosis; AVA, aortic valve area; AVC, aortic valve calcium score; DVI, Doppler velocity index; MDCT, multidetector computed tomography; MG, mean transvalvular gradient; SV, stroke volume, LVOT, left ventricular outflow tract, Q mean, mean transvalvular flow rate, Vmax, peak aortic velocity.

**Figure 6:**
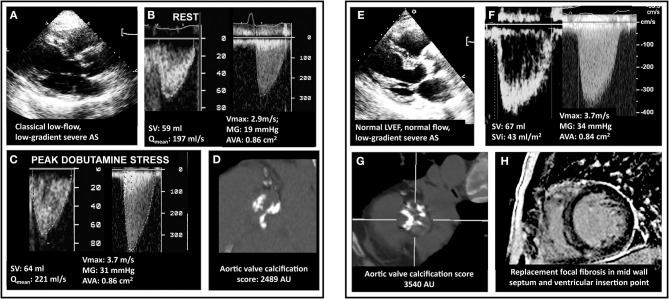
Multimodality imaging assessment of discordant low-gradient aortic stenosis. In **(A)**, a 75 year-old man with the classical form of low-flow, low-gradient AS i.e. with a left ventricular ejection fraction of 30%. The patient had an aortic valve area (AVA) <1.0, in discordance with a mean pressure gradient (MG) <40 mmHg **(B)**. Usually, dobutamine stress echocardiography allows to assess MG/AVA at flow normalizaion. However, as shown in **(C)**, and as happens in 30–40% of the patients, the discordance persisted, which was due to a minimal increase in transvalvular flow (Qmean). In these cases, it is recommended to measure the aortic valve calcification following the Agatston method and using sex-specific cutpoints (1200 AU for women and 2000 AU for men). This eventually allowed to confirm stenosis severity **(D)**. In **(E)**, a woman with mild symptoms (NYHA I-II) and discordant a priori severe AS but normal LVEF and normal stroke volume **(F)**. The AVC score is the primary approach in this subset of patients, as illustrated in the present case (**G** and confirmation of stenosis severity). Patients with normal LVEF are at lower risk than CLF patients. However, risk-stratification can be achieved using gadolinium enhanced cardiac magnetic resonance or NTproBNP. This patient exhibited focal myocardial fibrosis on CMR **(H)**. Also, her NT-proBNP was measured at 660 pg/ml i.e., 7-fold the upper reference level for age and sex. Both results indicate a high-risk profile and suggest that aortic valve replacement is a reasonable option.

Due to the aforementioned limitations of DSE, its small (but real) risks and the fact that MDCT AVC quantification is a low-radiation, quantitative and extremely reproducible technique, calcium scoring should be the method of choice to resolve discordant grading (for classical low-flow, paradoxical low-flow and normal-flow low-gradient AS) (Figures 4–6).

Limited experience exists with measurement of aortic valve calcium load on contrast enhanced images. The degree of opacification in contrast-enhanced CTs is the result of a complex interaction of multiple variables related to patient (body size, cardiac output), scanner (scan delay and duration, radiation dose) and contrast material (iodine concentration and volume, injection rate, bolus shape). Therefore, the thresholds for calcium detection on contrast-enhanced CT scans has not been standardized in the literature (74) and none of the studies has included prognostic information. The current evidence favors the use of non-contrast acquisitions for stenosis severity assessment using AVC measurement.

However, contrast-CT angiography is essential for transcatheter aortic valve replacement planning to define the sizing of the prosthesis according to annulus dimensions, aortic valve morphology (bicuspid vs. tricuspid), aortic dimensions, coronary height and peripheral vasculature (75).

The use of hybrid methods has been proposed by many researchers to resolve the discordant grading conundrum (76): it generally involves measuring LVOT area by a 3D method such as MDCT, CMR or 3D-TEE and combine it with Doppler-TTE estimation of LVOT VTI to calculate SV and aortic valve VTI to calculate AVA. However, they have not shown to be superior to echocardiography in predicting outcomes (46). If used, it must be noted that the cut-point for definition of severe AS (i.e., associated with increased mortality) using hybrid methods should be <1.2 cm^2^ and not <1 cm^2^ (46). A recent paper using hybrid method with CT LVOT area and Doppler VTI even demonstrated larger effective orifice areas than geometric orifice areas (77), which is incompatible with fluid mechanics of stenotic flow (78). Therefore, though TTE unequivocally underestimates LVOT area, it does not seem to underestimate stroke volume and/or AVA as compared to phase-contrast CMR (16, 27) or invasive methods (50). A potential physical explanation is that continuity equation assumes a relatively flat flow velocity profile (i.e., mean velocity equals peak velocity) with homogeneous distribution of velocities through the LVOT area. However, there is evidence that flow velocity profile along the LVOT is indeed not flat but often skewed, with higher velocities along the anterior and right aspects (27). Thus, the aforementioned LVOT area underestimation by TTE might be somewhat counterbalanced by a Doppler overestimation of LVOT VTI. This hypothesis provides a theoretical framework to explain the overestimation of AVA and SV by hybrid methods, which should be approached cautiously in order to not underestimate the severity of AS (79).

### Other Modalities

Use of CMR phase-contrast to assess AS severity is challenging and -mainly due to partial volume averaging- leads to significant underestimation of peak aortic velocity and VTI, and thus, AVA and AS severity (16, 80, 81). A recent meta-analysis showed that planimetry (geometric orifice area) by CMR correlated well with TEE, but were up to 11% larger than AVA estimated by TTE (81). However, the methods used in CMR were heterogeneous (planimetric, hybrid and effective orifice area) and there are known differences between the effective and geometric orifice area due to flow contraction at the vena contracta. Therefore, we believe CMR is not yet a method of choice for hemodynamic severity assessment in clinical practice. There might be a promising role for CMR 4D-flow (82–84), which can assess not only velocities (with the potential advantage of less underestimation than standard 2D through plane phase-contrast CMR), but also pressure and energy losses along the stenosis and into the aorta, and assessment of shear stress. Four-dimensional flow, however, still remains experimental and is not easily applicable to routine clinical practice due to long post-processing times. CMR is, however, an excellent technique for assessing aortic valve morphology, the aorta and quantifying of aortic regurgitation in native and prosthetic valves.

Table 1 presents a summary of imaging markers of AS severity and their cut-offs.

## Assessment of Myocardial Consequences of Aortic Stenosis

AS is a disease of both the valve and the myocardium (3). The myocardial response of the left ventricle to AS has a major impact on the presence of symptoms and clinical outcomes. Advanced cardiac imaging has greatly contributed to the understanding of the complex interaction between the increased afterload (both valvular due to AS and vascular due to increased peripheral resistance and aortic impedance) and the myocardial consequences (LV hypertrophy, adverse remodeling, myocardial fibrosis and, ultimately, decreased contractility) (4). However, the amount on which decreased LVEF is secondary to increased afterload (“*afterload mismatch,”* therefore relieved by AVR) or to intrinsic myocardial damage has proven to be difficult to assert. Table 2 presents multiple imaging markers that are used to assess myocardial impairment in patients with AS.

**Table 2:** Imaging markers to assess the myocardial consequences of aortic stenosis.

**Imaging modality**	**Cut-off for increased risk^a^**	**Concept**	**Advantages**	**Limitations**
**Rest echocardiography**				
LVEF (85, 86)	<50%^b^ (<60%^*c*^)	Percentage of LV volume ejected per beat	Universally used Extensive evidence on outcome prediction	Highly load-dependent (e.g., overestimation of pump function in significant mitral regurgitation) Insensitive to myocardial impairment in concentric remodeling/hypertrophy (frequent in AS) Guidelines' threshold of 50% already too low
Myocardial strain (87)	>−14.7%	Dimensionless index of myocardial deformation in 3 axes (longitudinal, radial and circumferential)	Less (but still significant) load-dependence vs. LVEF Earlier marker of “subclinical” myocardial impairment Predicts adverse outcomes	Reproducibility only clinically acceptable for global longitudinal strain Vendor-dependent results Feasibility dependent on echogenicity
Stroke volume index (13, 88, 89)	<35 ml/m^2^ (♀ <32 ml/m^2^ ♂ <40 ml/m^2^)	Volume of blood ejected by the LV adjusted for BSA	Good surrogate marker of LV pump function Directly related to cardiac output Incremental value vs. LVEF for risk prediction Extensive evidence on outcome prediction	LVOT area (required for SV calculation) particularly subject to measurement errors/geometric assumptions Standard definition does not consider gender differences Low-flow overestimated in obese patients Influenced by heart rate
Mean transvalvular flow rate (12)	<200 ml/s	Actual volumetric flow across the AV *(stroke volume/ejection time)*	Direct correlation with transvalvular gradients Adjusted for heart rate Potential incremental value vs. SVi	Does not take into account BSA More limited evidence than SVi
Cardiac damage staging system (5, 90)	≥ Stage 2	4-stage classification system characterizing the extent of extra-aortic valve cardiac damage	Systematic, holistic approach to whole-cardiac damage induced by (or coexistent with) AS Predicts outcome in symptomatic and asymptomatic AS and after TAVR	Does not distinguish between damage specifically caused by AS or comorbidity
**Dobutamine stress echocardiography**				
Contractile/flow reserve (67)	Increase in SV <20%	Capacity to increase SV with low-dose (20 ug/kg/min) associated with less severe myocardial impairment/myocardial viability	Historical data on outcome prediction Allows to distinguish pseudo-severe vs. true-severe AS Influenced by loading conditions (i.e., AS hemodynamic severity)	AVA_Proj_ outperforms flow reserve on evaluation of true-severe AS Does not change management (e.g., patients with classical LF-LG AS should undergo AVR regardless of flow reserve) Does not correlate with myocardial fibrosis measured by CMR (10)
**Cardiovascular magnetic resonance**				
Late gadolinium enhancement (91)	LGE (+) (non-infarct/infarct pattern) ↑ risk with ↑ fibrosis burden	Surrogate of focal (replacement) myocardial fibrosis	Both presence (LGE + vs. –) and quantity (fibrosis burden) associated with worse prognosis RCT undergoing (EVoLVeD) Allows precise tissue characterization to r/o concomitant diseases (e.g., myocardial infarction, cardiac amyloidosis)	Indicates irreversible damage (disease already too advanced?)
T1 mapping (92)	↑ risk with ↑ ECV (≥25.9%)	ECV surrogate marker of diffuse (reactive) fibrosis	More sensitive than LGE Allows precise tissue characterization to r/o concomitant diseases	No clinically valid threshold
Myocardial strain (93)	>−18%	Same as above	No echogenicity limitations No contrast required	Less spatiotemporal resolution than echo Cost and limited availability Limited evidence
**Multidetector computed tomography**				
Myocardial strain (94)	>−20.5%	Same as above	No echogenicity limitations	Poor spatiotemporal resolution Iodinated contrast required Limited evidence

*LVEF, left ventricular ejection fraction*.

a *Defined as the threshold associated with worse clinical outcomes*.

b *Guidelines' threshold*.

c *Recently proposed threshold (85, 86)*.

*AV, aortic valve, AVA, aortic valve area, EOA, effective orifice area; GOA, geometric orifice area; LVOT, left ventricular outflow tract*.

### Echocardiographic Markers of LV Impairment

Though markedly influenced by both preload and afterload, LVEF continues to be the most frequently used parameter to assess myocardial status. “Preserved” LVEF is defined in guidelines as ≥50% (7, 8, 37), though there is growing evidence of an increased mortality risk in patients with AS and an LVEF of 50–59% (85, 86). Most patients with AS and, especially those with paradoxical low-flow low-gradient AS have small cavities with increased wall thickness in which LVEF remains for long within normal range albeit a decreased longitudinal fiber contraction and a decreased stroke volume. This is also true in patients with left ventricular hypertrophy and especially cardiac amyloidosis, in which LVEF can remain ≥50% even until advanced stages of the disease (17). This reinforces the urge to revisit the current guideline threshold of an LVEF of 50% to define AVR indication (7, 8), as the risk increase appears to be more a continuum amongst the LVEF range than a sudden jump from “preserved” (>50%) and “depressed” (<50%) LVEF. However, this hypothesis warrants further study.

In order to overcome the limitations of LVEF and considering that subendocardial longitudinal fibers are affected early in the pathophysiology of pressure overload of AS, myocardial strain has emerged as a valuable imaging biomarker in AS. Due to its reproducibility and the fact that detects early changes in longitudinal function, global longitudinal strain (GLS) is the most reliable strain parameter and the only one that can be confidently incorporated into clinical practice (87, 95) for risk stratification and eventually consideration for early intervention among asymptomatic AS patients. Due to its high temporal resolution, almost universal availability and large body of evidence, TTE speckle tracking remains the most widespread imaging technique for deformation assessment. A recent meta-analysis of 10 studies including 1067 asymptomatic patients with AS, LVEF >50% identified a median LV GLS of −16.2% [−5.6 to −30.1%]. LV GLS showed modest predictive value for overall mortality (area under the curve 0.68) with an optimal cut-off of −14.7% (sensitivity 60%, specificity 70%) (87). More importantly, the relationship between LV GLS and mortality remained significant in patients with LVEF ≥60%, making GLS a potentially important imaging marker to trigger early intervention in asymptomatic patients. In the clinical setting, however, the relatively poor reproducibility and inter-vendor differences preclude its widespread use in clinical routine (95). Assessment of GLS can also be done by CMR, either by myocardial tagging (a specific sequence that imposes and follows planes of saturated myocardial magnetization) or feature tracking (which utilizes endocardial contour details along myocardial wall in routine cine SSFP image), but very few studies tested the prognostic value (93) with a proposed cut-off of < −18%. Interestingly, a recent study by Fukui et al. (94) showed the feasibility of GLS assessment by MDCT (with retrospective gating) and a reduced GLS (cut-off ≤ 20.5%) was independently associated with increased mortality post-transcatheter AVR.

Left ventricular stroke volume is the result of the interaction between preload, afterload and contractility and, along with heart rate, is the major determinant of cardiac output. It is, therefore, an excellent (albeit non-specific) surrogate of the overall LV function as a pump. Assessment of flow status (in practice, measurement of the stroke volume index [SVi] using Doppler echocardiography) has become paramount in the assessment of discordant grading AS, especially in those with low-gradient (i.e., an AVA <1 cm^2^ with a MG <40 mmHg) (7, 15). The impact of low-flow (i.e., SVi <35 ml/m^2^) on outcomes has been extensively demonstrated in patients with paradoxical low-gradient severe AS, both under medical management and after surgical or transcatheter aortic valve replacement (TAVR) (15, 96, 97). The consequences of a low-flow state in patients with high-gradient (HG) and preserved LVEF was, until recently, much less clear. In fact, after a high gradient has been established, severe AS is usually presumed and further evaluation of flow status (and according to some authors even calculation of AVA) is deemed unnecessary (7, 98, 99). However, there is growing evidence (and a sound pathophysiological reason) that SVi is a powerful predictor of outcomes even in patients with severe AS and a MG >40 mmHg (13, 88, 89). Therefore, we believe that SVi is a powerful independent prognostic factor that should be incorporated into routine pre-intervention risk scores and clinical decision making, even in patients with high gradient (100) (Figure 5). Taking into account sex-differences in left ventricular volumes and remodeling (women having more concentric remodeling and smaller LV cavity), we recently showed that the use of sex-specific thresholds (women: <32 ml/m^2^ and men <40 ml/m^2^) improved prognostic assessment in 1492 patients undergoing surgical AVR (13). These sex-specific thresholds should be further validated in independent, asymptomatic and transcatheter AVR cohorts, but they emphasize the need of sex-specific approaches in valvular heart disease.

Transvalvular mean flow rate (stroke volume/ejection time) is a useful parameter, especially in cases with normal “flow” (i.e., SVi ≥35 ml/m^2^) and low gradient (11, 101). A flow rate <200 ml/s has been shown to provide incremental prognostic value beyond clinical risk factors, LVEF and even SVi (12) in a population of severe AS patients undergoing AVR.

**Figure 7:**
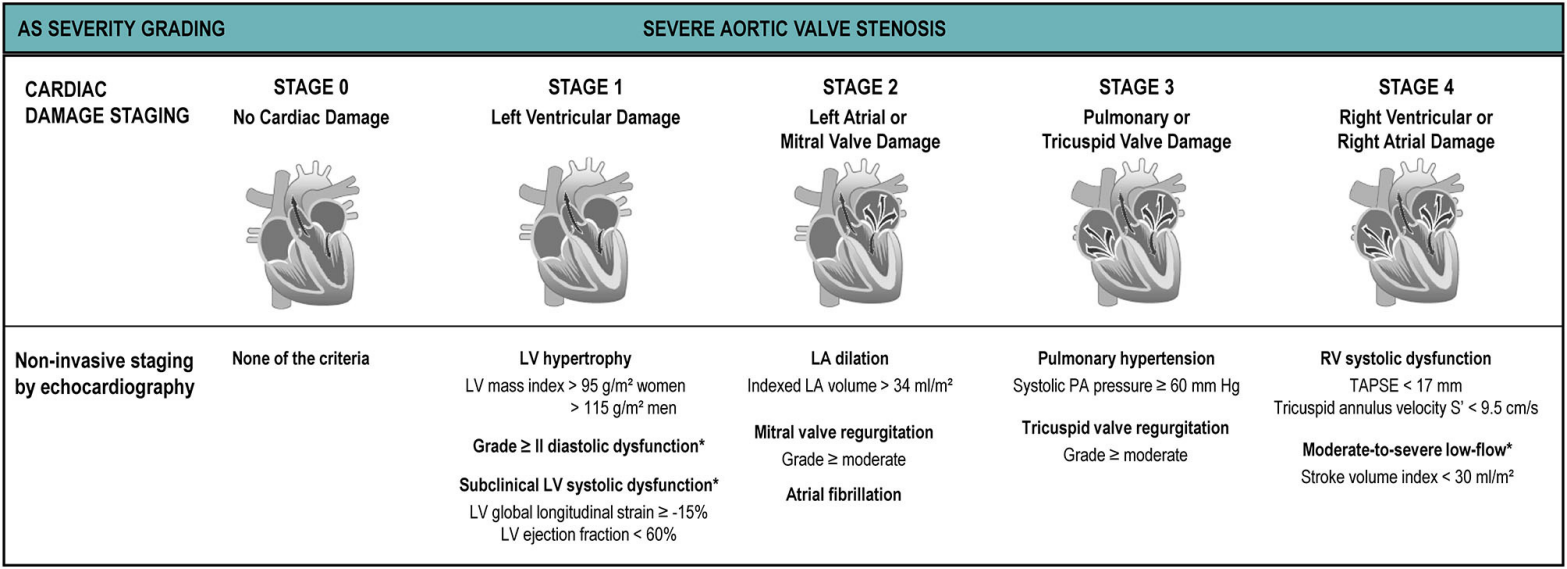
Cardiac damage staging system. The proposed cardiac damage staging scheme originally proposed by Généreux et al. (6) and modified by Tastet et al. (5) is based on a multi-parameter approach using echocardiographic parameters. The patient qualifies for a given stage if at least one of the proposed criteria for this stage is met. *Parameters added or modified by Tastet et al. to the original Génereux scheme for staging in asymptomatic patients with moderate or severe AS.

Besides LV systolic dysfunction and low-flow, a myriad of adverse prognostic factors have been described for patients with AS such as diastolic dysfunction, mitral regurgitation, pulmonary hypertension and right ventricular dysfunction. Recently, Généreux et al. have proposed a cardiac damage staging scheme based on echocardiographic parameters (6) (Figure 7): Stage 0, no cardiac damage; Stage 1: LV damage (LV hypertrophy, diastolic and/or systolic dysfunction); Stage 2: left atrial or mitral valve damage (left atrial dilation, atrial fibrillation and/or ≥moderate mitral regurgitation); Stage 3: pulmonary vasculature or tricuspid damage (systolic pulmonary hypertension and/or ≥moderate tricuspid regurgitation; and Stage 4: right ventricular damage (≥moderate RV systolic dysfunction). This staging system relies on the concept that increased LV afterload induced by the stenotic aortic valve leads to progressive and sequential damage to heart chambers in an upstream fashion. Even though it does not discriminate causality (e.g., a Stage 3 significant pulmonary hypertension can be secondary either to a very severe AS or to chronic obstructive pulmonary disease), it has proven to be a powerful predictor of mortality not only after AVR (6, 102) but also in symptomatic (90) and asymptomatic (5) AS patients. The latter used a modified schema suggested by Tastet et al. that incorporates some valuable changes, such as raise the severity cut-point of LVEF from 50% to 60% and incorporate GLS (<15%) as a mean to increase detection of subclinical LV dysfunction. The inclusion of moderate/severe low-flow (SVi <30 ml/m^2^) to Stage 4 further emphasizes the value of a decreased SVi as a surrogate of “subclinical heart failure” (Figure 7). The staging system provides a simple, useful framework to summarize cardiac damage secondary to (or concomitant with) valvular disease in AS patients using only TTE that can be easily incorporated into clinical practice.

Historically, flow reserve (an increase of ≥20% in stroke volume with low-dose DSE) has been considered a sign of “contractile reserve” in classical low-flow low-gradient AS and its absence, therefore, a marker of intrinsic myocardial damage and increased postoperative mortality (103, 104). The flow-reserve paradigm, however, has been recently challenged (67) as it does not provide a useful framework for decision making, as AVR is indicated nonetheless (Class I if flow reserve is present, Class IIa if absent). Moreover, the prognosis value of flow-reserve has not been reproduced in independent studies after surgical or transcatheter AVR (34, 105, 106). Finally, the quantity of diffuse interstitial fibrosis (by assessing extracellular volume [ECV] using T1 mapping techniques) and focal replacement fibrosis (by assessing late gadolinium enhancement [LGE]) in 41 patients with classical low-flow low-gradient AS were found similar in patients with and without flow reserve (albeit higher than in patients with high gradient AS) (10). Therefore, they further confirmed that patients with classical low-flow low-gradient have intrinsic myocardial damage as compared to high gradient AS but, more interestingly, they disproved the theory that patients without flow-reserve have more fibrosis than patients with flow reserve.

### Myocardial Fibrosis at CMR

It has been known for long that myocardial fibrosis was closely related to the transition from asymptomatic state to heart failure (107). In the last years CMR proved to be an invaluable tool for characterizing myocardial tissue (using LGE as a marker of focal fibrosis and T1 mapping/ECV calculation as a surrogate marker of diffuse fibrosis) and it has provided a unique opportunity to deepen our knowledge of the myocardial consequences of AS. The presence of LGE has been shown to be associated not only with adverse remodeling (108, 109) but also with increased mortality after AVR (110). A recent meta-analysis included six studies with 1151 patients, showing that LGE was present in 49% of patients with AS (most frequently men, of older age, diabetic and with decreased LVEF). Interestingly, no difference was found in AS severity (AVA) between patients with and without LGE, underscoring that AS severity and myocardial damage have a complex, non-linear relationship. LGE was found to be a strong predictor of mortality (adjusted hazard ratio 2.50, confidence interval 1.64 to 3.83) (91), further emphasizing its potential role as a marker for risk stratification and to optimize timing of AVR.

Diffuse myocardial fibrosis is an attractive biomarker, as it is somewhat reversible after AVR (108) and generally precedes irreversible focal fibrosis. ECV quantification by T1 mapping is a good−albeit imperfect−surrogate for diffuse fibrosis [especially in AS, where fibrosis follows a subendocardial gradient (109) and T1 mapping techniques have difficulties assessing the subendocardium due to partial volume effects in the blood-myocardial interphase (4)]. However, its reproducibility is lower than LGE techniques and, though center and scanner-specific cut-points exist, universal ECV/native T1 cut-points to guide clinical decision-making (and eventually be incorporated into guidelines) are yet to be confirmed. However, T1 mapping remains an invaluable tool for research and provides useful complementary information in clinical settings. Interestingly, in patients with similar LGE and AS severity, women appeared to have more ECV than men (111). After AVR, the extent of extracellular matrix decreases (in parallel with reductions in mass, albeit a slightly increased ECV), but the amount of scarring on LGE does not (108), indicating irreversible damage (analog to what was historically shown using myocardial biopsies). Therefore, there is a growing interest in using CMR as an imaging biomarker of early subclinical myocardial damage as a potential trigger for early intervention. This hypothesis is currently being tested in a randomized controlled trial (EVoLVeD, NCT03094143) (112).

### Multimodality Imaging in AS: A Practical Approach

Even though advanced non-invasive imaging has made huge advances in the understanding of the complex interaction between AS and the myocardial response and provides an invaluable framework for future research, a patient-centered (as opposed to imaging method-centered), practical approach should be emphasized in routine clinical practice. The fundamental question is not which imaging modality is best, but rather which imaging strategy is best suited for a given patient and/or clinical scenario.

The EACVI survey provides an interesting snapshot of contemporary routine clinical practice (albeit in university, high-volume centers) (41): only 52% of centers used right parasternal view to assess peak velocity and gradient measurements, AVA was not even calculated in 7% of centers and 45% of them did not assess flow status (though SV calculation is intrinsically necessary for AVA calculation using continuity equation) and 52% did not calculate dimensionless index. Furthermore, one third of centers did not routinely assess blood pressure. Therefore, before moving on to more advanced imaging techniques, it is paramount to optimize TTE, which remains (and will likely remain for a long time) the cornerstone of AS assessment, as it allows a comprehensive evaluation of both valvular disease and its impact on the myocardium (including staging of cardiac damage).

Indeed, a majority of patients will have concordant hemodynamic parameters and TTE will be conclusive. Interestingly, in the EACVI survey, in cases with discordant grading the majority of centers carefully reviewed the original TTE scans. In paradoxical low-flow low-gradient AS, two thirds of centers performed an MDCT AVC quantification, as per recommendation of ESC/EACTS guidelines (7). In classical low-flow low-gradient AS, DSE was more frequently used, but still more than 40% of centers performed AVC quantification. Valve morphology was most frequently assessed by TEE or 3D-TEE, but CMR and MDCT were also considered. CMR was used to corroborate stroke volume (and flow-status) in 25% of centers.

Our suggested approach to AS is summarized in the algorithm proposed in Figure 5. AVA should be calculated in both high gradient (to rule out high flow states such as anemia, hyperthyroidism or other causes of high cardiac output) and low gradient (to rule out moderate AS). Flow status (by SVi and/or flow rate) should be assessed systematically, as it is of prognostic value not only in low gradient but also in those with a MG ≥40 mmHg. In confirmed (after careful corroboration of TTE measurements) discordant grading patients (i.e., AVA <1 cm^2^ and/or indexed AVA ≤ 0.6 cm^2^/m^2^ with a MG <40 mmHg and Vpeak <4 m/s), we believe MDCT AVC scoring should be the next method of choice, as it is provides a flow-independent assessment of anatomic severity and has proven to be a powerful predictor of clinical outcomes. The potential for adverse effects (though infrequent), the relatively high frequency of non-conclusive results and the fact that flow reserve is being increasingly challenged downgrades DSE as a third alternative after MDCT for AS severity adjudication. CMR, especially with the use of tissue characterization techniques (LGE and T1 mapping/ECV) and the use of blood biomarkers such as N-terminal Pro b-type Natriuretic Peptide allows an excellent characterization of myocardial damage that may help guide the timing of intervention and improve risk stratification. Use of MDCT and/or TEE for transcatheter AVR planning is paramount, as well as the use of CMR and/or MDCT to assess valve morphology and aortic diameters in bicuspid AS patients. Exercise testing with echocardiography may be useful in allegedly asymptomatic patients to ascertain symptomatic status and assess AS severity in paradoxical low flow patients.

## Conclusion

Even though advanced cardiovascular imaging is here to stay and its applications to valvular heart disease (and specifically to AS) continue to expand, at a population level there remains a vast percentage of patients with AS who are not recognized and/or never referred for evaluation (113, 114). Therefore, an effort should be undergone to improve screening and increase access to Doppler TTE, which remains the most cost-effective imaging technique and the cornerstone of AS diagnosis and management. In a high proportion of patients, a meticulous approach (including assessment of flow-status, calculation of AVA, right parasternal window, cardiac damage staging and careful assessment of LVEF/GLS) will provide the answer to the severity of the stenosis and the status of the myocardium. In the percentage (30 to 40%) that remains challenging, MDCT AVC quantification should be performed. Usefulness of CMR-LGE as a trigger for early surgical AVR has shown great potential and is currently being tested in a randomized controlled trial (EVoLVeD).

## Author Contributions

EG and M-AC wrote the first draft of the manuscript, figures, and tables. M-SA helped for figure and case preparation. M-SA, PP, and M-AC made a careful review of the manuscript. All authors contributed to the article and approved the submitted version.

## Conflict of Interest

The authors declare that the research was conducted in the absence of any commercial or financial relationships that could be construed as a potential conflict of interest.

## Abbreviations

AS: aortic stenosis
AVA: aortic valve area
AVC: aortic valve calcium
AVR: aortic valve replacement
CMR: cardiovascular magnetic resonance
ECV: extracellular volume
GLS: global longitudinal strain
LV: left ventricle
MDCT: multidetector computed tomography
SVi: stroke volume index
TTE: transthoracic Doppler-echocardiography
VTI: velocity-time integral.

## References

[1] Clavel MA Burwash IG Pibarot P Cardiac imaging for assessing low-gradient severe aortic stenosis. JACC Cardiovasc Imaging. (2017) 10:185–202. 10.1016/j.jcmg.2017.01.002 28183438

[2] Berthelot-Richer M Pibarot P Capoulade R Dumesnil JG Dahou A Thébault C. Discordant grading of aortic stenosis severity: echocardiographic predictors of survival benefit associated with aortic valve replacement. JACC Cardiovasc Imaging. (2016) 9:797–805. 10.1016/j.jcmg.2015.09.026 27209111

[3] Dweck MR Boon NA Newby DE. Calcific aortic stenosis: a disease of the valve and the myocardium. J Am Coll Cardiol. (2012) 60:1854–63. 10.1016/j.jacc.2012.02.093 23062541

[4] Treibel TA Badiani S Lloyd G Moon JC. Multimodality imaging markers of adverse myocardial remodeling in aortic stenosis. JACC Cardiovasc Imaging. (2019) 12:1532–48. 10.1016/j.jcmg.2019.02.034 31395243

[5] Tastet L Tribouilloy C Maréchaux S Vollema EM Delgado V Salaun E. Staging cardiac damage in patients with asymptomatic aortic valve stenosis. J Am Coll Cardiol. (2019) 74:550–63. 10.1016/j.jacc.2019.04.065 31345430

[6] Généreux P Pibarot P Redfors B Mack MJ Makkar RR Jaber WA. Staging classification of aortic stenosis based on the extent of cardiac damage. Eur Heart J. (2017) 74:550–63. 10.1093/eurheartj/ehx381 29020232 PMC5837727

[7] Baumgartner H Falk V Bax JJ de Bonis M Hamm C Holm PJ 2017 ESC/EACTS guidelines for the management of valvular heart disease: the task force for the management of valvular heart disease of the European society of cardiology (ESC) and the European association for cardio-thoracic surgery (EACTS). Eur Heart J. (2017) 38:2739–91. 10.5603/KP.2018.0013 28886619

[8] Nishimura RA Otto CM Bonow RO Carabello BA Erwin JP III Guyton RA O'Gara PT 2014 AHA/ACC guideline for the management of patients with valvular heart disease: a report of the American college of cardiology/American heart association task force on practice guidelines. J Am Coll Cardiol. (2014) 63:e57–185. 10.1161/CIR.0000000000000029 24603191

[9] Urena M Mok M Serra V Dumont É Nombela-Franco L DeLarochellière R. Predictive factors and long-term clinical consequences of persistent left bundle branch block following transcatheter aortic valve implantation with a balloon-expandable valve. J Am Coll Cardiol. (2012) 60:1743–52. 10.1016/j.jacc.2012.07.035 23040577

[10] Rosa VEE Ribeiro HB Sampaio RO Morais TC Rosa MEE Pires LJT. Myocardial fibrosis in classical low-flow, low-gradient aortic stenosis. Circ Cardiovasc Imaging. (2019) 12:e008353. 10.1161/CIRCIMAGING.118.008353 31088148

[11] Shah BN Chahal NS Senior R. Low-flow low-gradient aortic stenosis in patients with low ejection fraction: but is the flow truly low? Int J Cardiol. (2013) 168:4999–5001. 10.1016/j.ijcard.2013.07.125 23915521

[12] Vamvakidou A Jin W Danylenko O Pradhan J Li W West C. Impact of pre-intervention transaortic flow rate versus stroke volume index on mortality across the hemodynamic spectrum of severe aortic stenosis: implications for a new hemodynamic classification of aortic stenosis. JACC Cardiovasc Imaging. (2019) 12:205–6. 10.1016/j.jcmg.2018.11.004 30621992

[13] Guzzetti E Poulin A Annabi MS Zhang B Kalavrouziotis D Couture C. Transvalvular flow, sex, and survival after valve replacement surgery in patients with severe aortic stenosis. J Am Coll Cardiol. (2020) 75:1897–909. 10.1016/j.jacc.2020.02.065 32327100

[14] Annabi MS Clisson M Clavel MA Pibarot P. Workup and management of patients with paradoxical low-flow, low-gradient aortic stenosis. Curr Treat Options Cardiovasc Med. (2018) 20:49. 10.1007/s11936-018-0642-y 29721704

[15] Clavel MA Magne J Pibarot P. Low-gradient aortic stenosis. Eur Heart J. (2016) 37:2645–57. 10.1093/eurheartj/ehw096 27190103 PMC5030681

[16] Guzzetti E Capoulade R Tastet L Garcia J Le Ven F Arsenault M. Estimation of stroke volume and aortic valve area in patients with aortic stenosis: a comparison of echocardiography versus cardiovascular magnetic resonance. J Am Soc Echocardiogr. (2020) 33:953–63.e5. 10.1016/j.echo.2020.03.020 32580897

[17] Ternacle J Krapf L Mohty D Magne J Nguyen A Galat A. Aortic stenosis and cardiac amyloidosis: JACC review topic of the week. J Am Coll Cardiol. (2019) 74:2638–51. 10.1016/j.jacc.2019.09.056 31753206

[18] Clavel M-A Guzzetti E Annabi M-S Salaun E Ong G Pibarot P Normal-flow low-gradient severe aortic stenosis: myth or reality? Struct Heart. (2018) 2:180–7. 10.1080/24748706.2018.1437934

[19] Zusman O Pressman GS Banai S Finkelstein A Topilsky Y. Intervention versus observation in symptomatic patients with normal flow-low gradient severe aortic stenosis. JACC Cardiovasc Imaging. (2017) 11:1225–32. 10.1016/j.jcmg.2017.07.020 29055632

[20] Lancellotti P Magne J Donal E Davin L O'Connor K Rosca M. Clinical outcome in asymptomatic severe aortic stenosis: insights from the new proposed aortic stenosis grading classification. J Am Coll Cardiol. (2012) 59:235–43. 10.1016/j.jacc.2011.08.072 22240128

[21] Minners J Allgeier M Gohlke-Baerwolf C Kienzle RP Neumann FJ Jander N. Inconsistencies of echocardiographic criteria for the grading of aortic valve stenosis. Eur Heart J. (2008) 29:1043–48. 10.1093/eurheartj/ehm543 18156619

[22] Côté N Simard L Zenses AS Tastet L Shen M Clisson M. Impact of vascular hemodynamics on aortic stenosis evaluation: new insights into the pathophysiology of normal flow-small aortic valve area-low gradient pattern. J Am Heart Assoc. (2017) 6:e006276. 10.1161/JAHA.117.006276 28687561 PMC5586319

[23] Otto CM. Mind the gap: missed valve disease diagnosis. Heart. (2018) 104:1810–1. 10.1136/heartjnl-2018-313474 29794243

[24] Pibarot P Sengupta P Chandrashekhar Y Imaging is the cornerstone of the management of aortic valve stenosis. JACC Cardiovasc Imaging. (2019) 12:220–3. 10.1016/j.jcmg.2018.12.001 30621995

[25] Pawade T Clavel MA Tribouilloy C Dreyfus J Mathieu T Tastet L. Computed tomography aortic valve calcium scoring in patients with aortic stenosis. Circ Cardiovasc Imaging. (2018) 11:e007146. 10.1161/CIRCIMAGING.117.007146 29555836

[26] Clavel MA Messika-Zeitoun D Pibarot P Aggarwal S Malouf J Araoz P. The complex nature of discordant severe calcified aortic valve disease grading: new insights from combined doppler-echocardiographic and computed tomographic study. J Am Coll Cardiol. (2013) 62:2329–38. 10.1016/j.jacc.2013.08.1621 24076528

[27] Garcia J Kadem L Larose É Clavel MA Pibarot P. Comparison between cardiovascular magnetic resonance imaging and transthoracic doppler echocardiography for the estimation of valve effective orifice area in patients with aortic stenosis. J Cardiovasc Magn Reson. (2011) 13:25. 10.1186/1532-429X-13-25 21527021 PMC3108925

[28] Dweck MR Joshi S Murigu T Gulati A Alpendurada F Jabbour A. Left ventricular remodeling and hypertrophy in patients with aortic stenosis: insights from cardiovascular magnetic resonance. J Cardiovasc Magn Reson. (2012) 14:50. 10.1186/1532-429X-14-50 22839417 PMC3457907

[29] Chambers JB Myerson SG Rajani R Morgan-Hughes GJ Dweck MR. Multimodality imaging in heart valve disease. Open Heart. (2016) 3:e000330. 10.1136/openhrt-2015-000330 26977308 PMC4785435

[30] Soon J Pibarot P Blanke P Ohana M Leipsic J. Multimodality imaging for planning and follow-up of transcatheter aortic valve replacement. Can J Cardiol. (2017) 33:1110–23. 10.1016/j.cjca.2017.03.024 28666614

[31] Dweck MR Jones C Joshi NV Fletcher AM Richardson H White A. Assessment of valvular calcification and inflammation by positron emission tomography in patients with aortic stenosis. Circulation. (2012) 125:76–86. 10.1161/CIRCULATIONAHA.111.051052 22090163

[32] Jenkins WS Vesey AT Shah AS Pawade TA Chin CW White AC. Valvular (18)F-fluoride and (18)F-fluorodeoxyglucose uptake predict disease progression and clinical outcome in patients with aortic stenosis. J Am Coll Cardiol. (2015) 66:1200–1. 10.1016/j.jacc.2015.06.1325 26338001

[33] Cartlidge TRG Doris MK Sellers SL Pawade TA White AC Pessotto R. Detection and prediction of bioprosthetic aortic valve degeneration. J Am Coll Cardiol. (2019) 73:1107–19. 10.1016/j.jacc.2018.12.056 30871693 PMC6424589

[34] Annabi MS Touboul E Dahou A Burwash IA Bergler-Klein J Enriquez-Sarano M. Dobutamine stress echocardiography for management of low-flow, low-gradient aortic stenosis. J Am Coll Cardiol. (2018) 71:475–85. 10.1016/j.jacc.2017.11.052 29406851

[35] Pawade T Sheth T Guzzetti E Dweck MR Clavel MA. Why and how to measure aortic valve calcification in patients with aortic stenosis. JACC Cardiovasc Imaging. (2019) 12:1835–48. 10.1016/j.jcmg.2019.01.045 31488252

[36] Clavel MA Pibarot P Messika-Zeitoun D Capoulade R Malouf J Aggarval S. Impact of aortic valve calcification, as measured by MDCT, on survival in patients with aortic stenosis: results of an international registry study. J Am Coll Cardiol. (2014) 64:1202–13. 10.1016/j.jacc.2014.05.066 25236511 PMC4391203

[37] Baumgartner H Hung J Bermejo J Chambers JB Edvardsen T Goldstein S. Recommendations on the echocardiographic assessment of aortic valve stenosis: a focused update from the European association of cardiovascular imaging and the American society of echocardiography. J Am Soc Echocardiogr. (2017) 30:372–92. 10.1093/ehjci/jew335 28385280

[38] Abbas AE Pibarot P. Hemodynamic characterization of aortic stenosis states. Catheter Cardiovasc Interv. (2019) 93:1002–23. 10.1002/ccd.28146 30790429

[39] de Monchy CC Lepage L Boutron I Leye M Detaint D Hyafil F. Usefulness of the right parasternal view and non-imaging continuous-wave doppler transducer for the evaluation of the severity of aortic stenosis in the modern area. Eur J Echocardiogr. (2009) 10:420–4. 10.1093/ejechocard/jen301 19036750

[40] Thaden JJ Nkomo VT Lee KJ Oh JK. Doppler Imaging in aortic stenosis: the importance of the nonapical imaging windows to determine severity in a contemporary cohort. J Am Soc Echocardiogr. (2015) 28:780–85. 10.1016/j.echo.2015.02.016 25857547

[41] Michalski B Dweck MR Marsan NA Cameli M D'Andrea A Carvalho RF. The evaluation of aortic stenosis, how the new guidelines are implemented across Europe: a survey by EACVI. Eur Heart J Cardiovasc Imaging. (2020) 21:357–62. 10.1093/ehjci/jeaa009 32196100

[42] Pibarot P Larose É What our eyes see is not necessarily what our heart feels. Cardiology. (2008) 109:122–5. 10.1159/000105553 17713327

[43] Hahn RT Pibarot P. Accurate measurement of left ventricular outflow tract diameter: comment on the updated recommendations for the echocardiographic assessment of aortic valve stenosis. J Am Soc Echocardiogr. (2017) 30:1038–41. 10.1016/j.echo.2017.06.002 28764864

[44] Mehrotra P Flynn AW Jansen K Tan TC Mak G Julien HM. Differential left ventricular outflow tract remodeling and dynamics in aortic stenosis. J Am Soc Echocardiogr. (2015) 28:1259–66. 10.1016/j.echo.2015.07.018 26307374

[45] Caballero L Saura D Oliva-Sandoval MJ Gonzalez-Carrillo J Espinosa MD Garcia-Navarro M. Three-dimensional morphology of the left ventricular outflow tract: impact on grading aortic stenosis severity. J Am Soc Echocardiogr. (2017) 30:28–35. 10.1016/j.echo.2016.10.006 27887818

[46] Clavel MA Malouf J Messika-Zeitoun D Araoz PA Michelena HI Enriquez-Sarano M. Aortic valve area calculation in aortic stenosis by CT and doppler echocardiography. JACC Cardiovasc Imaging. (2015) 8:248–57. 10.1016/j.jcmg.2015.01.009 25772832

[47] Chin CW Khaw HJ Luo E Tan S White AC Newby DE. Echocardiography underestimates stroke volume and aortic valve area: implications for patients with small-area low-gradient aortic stenosis. Can J Cardiol. (2014) 30:1064–72. 10.1016/j.cjca.2014.04.021 25151288 PMC4161727

[48] Maes F Pierard S de Meester C Boulif J Amzulescu M vancraeynest D. Impact of left ventricular outflow tract ellipticity on the grading of aortic stenosis in patients with normal ejection fraction. J Cardiovasc Magn Reson. (2017) 19:37. 10.1186/s12968-017-0344-8 28292302 PMC5351048

[49] Stahli BE Stadler T Holy EW Nguyen-Kim TDL Hoffelner L Erhart L. Impact of stroke volume assessment by integrating multi-detector computed tomography and doppler data on the classification of aortic stenosis. Int J Cardiol. (2017) 246:80–6. 10.1016/j.ijcard.2017.03.112 28867024

[50] LaBounty TM Miyasaka R Chetcuti S Grossman PM Deeb GM Patel HJ. Annulus instead of LVOT diameter improves agreement between echocardiography effective orifice area and invasive aortic valve area. JACC Cardiovasc Imaging. (2014) 7:1065–6. 10.1016/j.jcmg.2014.03.021 25323170

[51] Zoghbi WA. Velocity acceleration in aortic stenosis revisited. JACC Cardiovasc Imaging. (2015) 8:776–8. 10.1016/j.jcmg.2015.04.005 26183551

[52] Rusinaru D Malaquin D Maréchaux S Debry N Tribouilloy C. Relation of dimensionless index to long-term outcome in aortic stenosis with preserved LVEF. JACC. Cardiovasc. Imaging. (2015) 8:766–75. 10.1016/j.jcmg.2015.01.023 26093931

[53] Gamaza-Chulian S Diaz-Retamino E Camacho-Freire S Ruiz-Fernandez D Gutierrez-Barrios A Oneto-Otero J. Acceleration time and ratio of acceleration time to ejection time in aortic stenosis: new echocardiographic diagnostic parameters. J Am Soc Echocardiogr. (2017) 30:947–55. 10.1016/j.echo.2017.06.001 28781116

[54] Altes A Thellier N Bohbot Y Marsou W Chadha G Binda C. Prognostic impact of the ratio of acceleration time to ejection time in patients with low gradient severe aortic stenosis and preserved ejection fraction. Am J Cardiol. (2019) 124:1594–600. 10.1016/j.amjcard.2019.07.064 31522771

[55] Altes A Sochala M Attias D Dreyfus J Bohbot Y Toledano M. Correlates of the ratio of acceleration time to ejection time in patients with aortic stenosis: an echocardiographic and computed tomography study. Arch Cardiovasc Dis. (2019) 112:567–75. 10.1016/j.acvd.2019.06.004 31402281

[56] Gilon D Cape EG Handschumacher MD Song JK Solheim J VanAuker M Effect of three-dimensional valve shape on the hemodynamics of aortic stenosis: three-dimensional echocardiograpic stereolithography and patient studies. J Am Coll Cardiol. (2002) 40:1479–86. 10.1016/S0735-1097(02)02269-6 12392840

[57] Kadem L Rieu R Dumesnil JG Durand LG Pibarot P. Flow-dependent changes in doppler-derived aortic valve effective orifice area are real and not due to artifact. J Am Coll Cardiol. (2006) 47:131–7. 10.1016/j.jacc.2005.05.100 16386676

[58] Kim CJ Berglund H Nishioka T Luo H Siegel RJ. Correspondence of aortic valve area determination from transesophageal echocardiography, transthoracic echocardiography, cardiac catheterization. Am Heart J. (1996) 132:1163–72. 10.1016/S0002-8703(96)90459-7 8969567

[59] Akins CW Travis B Yoganathan AP. Energy loss for evaluating heart valve performance. J Thorac Cardiovasc Surg. (2008) 136:820–33. 10.1016/j.jtcvs.2007.12.059 18954618

[60] Garcia D Pibarot P Dumesnil JG Sakr F Durand LG. Assessment of aortic valve stenosis severity: a new index based on the energy loss concept. Circulation. (2000) 101:765–71. 10.1161/01.CIR.101.7.765 10683350

[61] Pibarot P Garcia D Dumesnil JG. Energy loss index in aortic stenosis: from fluid mechanics concept to clinical application. Circulation. (2013) 127:1101–4. 10.1161/CIRCULATIONAHA.113.001130 23479666

[62] Bahlmann E Gerdts E Cramariuc D Gohlke-Baerwolf C Nienaber CA Wachtell K. Prognostic value of energy loss index in asymptomatic aortic stenosis. Circulation. (2013) 127:1149–56. 10.1161/CIRCULATIONAHA.112.078857 23357717

[63] Spitzer E van Mieghem NM Pibarot P Hahn RT Kodali S Maurer MS. Rationale and design of the transcatheter aortic valve replacement to UNload the left ventricle in patients with ADvanced heart failure (TAVR UNLOAD) trial. Am Heart J. (2016) 182:80–8. 10.1016/j.ahj.2016.08.009 27914503

[64] Clavel MA Burwash IG Mundigler G Dumesnil JG Baumgartner H Bergler-Klein J. Validation of conventional and simplified methods to calculate projected valve area at normal flow rate in patients with low flow, low gradient aortic stenosis: the multicenter TOPAS (True or pseudo severe aortic stenosis) study. J Am Soc Echocardiogr. (2010) 23:380–6. 10.1016/j.echo.2010.02.002 20362927

[65] Fougères É Tribouilloy C Monchi M Petit-Eisenmann H Baleynaud S Pasquet A. Outcomes of pseudo-severe aortic stenosis under conservative treatment. Eur Heart J. (2012) 33:2426–33. 10.1093/eurheartj/ehs176 22733832

[66] Blais C Burwash IG Mundigler G Dumesnil JG Loho N Rader F. Projected valve area at normal flow rate improves the assessment of stenosis severity in patients with low flow, low-gradient aortic stenosis: the multicenter TOPAS (Truly or pseudo severe aortic stenosis) study. Circulation. (2006) 113:711–21. 10.1161/CIRCULATIONAHA.105.557678 16461844

[67] Annabi MS Clavel MA Pibarot P. Dobutamine stress echocardiography in low-flow, low-gradient aortic stenosis: flow reserve does not matter anymore. J Am Heart Assoc. (2019) 8:e012212. 10.1161/JAHA.119.012212 30879376 PMC6475035

[68] Clavel MA Ennezat PV Maréchaux S Dumesnil JG Capoulade R Hachicha Z. Stress echocardiography to assess stenosis severity and predict outcome in patients with paradoxical low-flow, low-gradient aortic stenosis and preserved LVEF. J Am Coll Cardiol Img. (2013) 6:175–83. 10.1016/j.jcmg.2012.10.015 23489531

[69] Guzzetti E Simard L Clisson M Clavel MA. Multiplanar “en face” reconstruction of the aortic valve: impact on aortic valve calcium scoring. JACC Cardiovasc Imaging. (2020). 10.1016/j.jcmg.2020.05.021. [Epub ahead of print]. 32739368

[70] Simard L Côté N Dagenais F Mathieu P Couture C Trahan S Sex-related discordance between aortic valve calcification and hemodynamic severity of aortic stenosis: is valvular fibrosis the explanation? Circ Res. (2017) 120:681–91. 10.1161/CIRCRESAHA.116.309306 27879282

[71] Patel DK Green KD Fudim M Harrell FE Wang TJ Robbins MA. Racial differences in the prevalence of severe aortic stenosis. J Am Heart Assoc. (2014) 3:e000879. 10.1161/JAHA.114.000879 24870936 PMC4309086

[72] Nasir K Katz R Takasu J Shavelle DM Detrano R Lima JA. Ethnic differences between extra-coronary measures on cardiac computed tomography: multi-ethnic study of atherosclerosis (MESA). Atherosclerosis. (2008) 198:104–14. 10.1016/j.atherosclerosis.2007.09.008 17950742 PMC11505142

[73] Guzzetti E Oh JK Shen M Dweck M Poh KK Abbas AE Ethnic differences in computed tomography aortic valve calcium quantification in patients with aortic stenosis. J Am Coll Cardiol. (2020) 75(Suppl.):2141 10.1016/S0735-1097(20)32768-6

[74] Bettinger N Khalique OK Krepp JM Hamid NB Bae DJ Pulerwitz TC. Practical determination of aortic valve calcium volume score on contrast-enhanced computed tomography prior to transcatheter aortic valve replacement and impact on paravalvular regurgitation: elucidating optimal threshold cutoffs. J Cardiovasc Comput Tomogr. (2017) 11:302–8. 10.1016/j.jcct.2017.04.009 28457950

[75] Blanke P Weir-McCall JR Achenbach S Delgado V Hausleiter J Jilaihawi H. Computed tomography imaging in the context of transcatheter aortic valve implantation (TAVI)/transcatheter aortic valve replacement (TAVR): an expert consensus document of the society of cardiovascular computed tomography. JACC Cardiovasc Imaging. (2019) 12:1–24. 10.1016/j.jcmg.2018.12.003 30621986

[76] Delgado V Clavel MA Hahn RT Gillam L Bax J Sengupta PP. How do we reconcile echocardiography, computed tomography, and hybrid imaging in assessing discordant grading of aortic stenosis severity? JACC Cardiovasc Imaging. (2019) 12:267–82. 10.1016/j.jcmg.2018.11.027 30732722

[77] Jander N Wienecke S Dorfs S Ruile P Neumann FJ Pache G. Anatomic estimation of aortic stenosis severity vs “fusion” of data from computed tomography and doppler echocardiography. Echocardiography. (2018) 35:777–84. 10.1111/echo.13855 29522643

[78] Machac J Bacharach SL Bateman TM Bax JJ Beanlands R Bengel F. Positron emission tomography myocardial perfusion and glucose metabolism imaging. J Nucl Cardiol. (2006) 13:e121–51. 10.1016/j.nuclcard.2006.08.009 17174789

[79] Pibarot P Clavel MA. Doppler echocardiographic quantitation of aortic valve stenosis: a science in constant evolution. J Am Soc Echocardiogr. (2016) 29:1019–22. 10.1016/j.echo.2016.08.015 27712803

[80] Karamitsos TD Myerson SG. The role of cardiovascular magnetic resonance in the evaluation of valve disease. Prog Cardiovasc Dis. (2011) 54:276–86. 10.1016/j.pcad.2011.08.005 22014494

[81] Woldendorp K Bannon PG Grieve SM. Evaluation of aortic stenosis using cardiovascular magnetic resonance: a systematic review & meta-analysis. J Cardiovasc Magn Reson. (2020) 22:45. 10.1186/s12968-020-00633-z 32536342 PMC7294634

[82] Garcia J Barker AJ Markl M. The role of imaging of flow patterns by 4D flow MRI in aortic stenosis. JACC Cardiovasc Imaging. (2019) 12:252–66. 10.1016/j.jcmg.2018.10.034 30732721

[83] Jamalidinan F Hassanabad AF Francois CJ Garcia J. Four-dimensional-flow magnetic resonance imaging of the aortic valve and thoracic aorta. Radiol Clin North Am. (2020) 58:753–63. 10.1016/j.rcl.2020.02.008 32471542

[84] Archer GT Elhawaz A Barker N Fidock B Rothman A van der Geest RJ. Validation of four-dimensional flow cardiovascular magnetic resonance for aortic stenosis assessment. Sci Rep. (2020) 10:10569. 10.1038/s41598-020-66659-6 32601326 PMC7324609

[85] Bohbot Y de Meester de Ravenstein C Chadha G Rusinaru D Belkhir K Trouillet C. Relationship between left ventricular ejection fraction and mortality in asymptomatic and minimally symptomatic patients with severe aortic stenosis. JACC Cardiovasc Imaging. (2019) 12:38–48. 10.1016/j.jcmg.2018.07.029 30448114

[86] Lancellotti P Magne J Dulgheru R Clavel MA Donal E Vannan MA Outcomes of patients with asymptomatic aortic stenosis followed up in heart valve clinics. JAMA Cardiol. (2018) 3:1060–68. 10.1001/jamacardio.2018.3152 30285058 PMC6583052

[87] Magne J Cosyns B Popescu BA Carstensen HG Dahl J Desai MY. Distribution and prognostic significance of left ventricular global longitudinal strain in asymptomatic significant aortic stenosis: an individual participant data meta-analysis. JACC Cardiovasc Imaging. (2019) 12:84–92. 10.1016/j.jcmg.2018.11.005 30621997

[88] Alkhalil M Brennan P McQuillan C Jeganathan R Manoharan G Owens CG. reflected by stroke volume index, is a risk marker in high-gradient aortic stenosis patients undergoing transcatheter aortic valve replacement. Can J Cardiol. (2020) 36:112–8. 10.1016/j.cjca.2019.08.030 31785992

[89] Marechaux S Rusinaru D Altes A Pasquet A Vanoverschelde JL Tribouilloy C. Prognostic value of low flow in patients with high transvalvular gradient severe aortic stenosis and preserved left ventricular ejection fraction: a multicenter study. Circ Cardiovasc Imaging. (2019) 12:e009299. 10.1161/CIRCIMAGING.119.009299 31597467

[90] Vollema EM Amanullah MR Ng ACT van der Bijl P Prevedello F Sin YK. Staging cardiac damage in patients with symptomatic aortic valve stenosis. J Am Coll Cardiol. (2019) 74:538–49. 10.1016/j.jacc.2019.05.048 31345429

[91] Papanastasiou CA Kokkinidis DG Kampaktsis PN Bikakis I Cunha DK Oikonomou EK. The prognostic role of late gadolinium enhancement in aortic stenosis: a systematic review and meta-analysis. JACC Cardiovasc Imaging. (2020) 13:385–92. 10.1016/j.jcmg.2019.03.029 31326491

[92] Everett RJ Treibel TA Fukui M Lee H Rigolli M Singh A. Extracellular myocardial volume in patients with aortic stenosis. J Am Coll Cardiol. (2020) 75:304–16. 10.1016/j.jacc.2019.11.032 31976869 PMC6985897

[93] Agoston-Coldea L Bheecarry K Cionca C Petra C Strimbu L Ober C. Incremental predictive value of longitudinal axis strain and late gadolinium enhancement using standard cmr imaging in patients with aortic stenosis. J Clin Med. (2019) 8:165. 10.3390/jcm8020165 30717180 PMC6406708

[94] Fukui M Xu J Thoma F Sultan I Mulukutla S Elzomor H. Baseline global longitudinal strain by computed tomography is associated with post transcatheter aortic valve replacement outcomes. J Cardiovasc Comput Tomogr. (2019) 14:233–9. 10.1016/S0735-1097(19)32072-8 31836414

[95] Voigt JU Cvijic M. 2- and 3-dimensional myocardial strain in cardiac health and disease. JACC Cardiovasc Imaging. (2019) 12:1849–63. 10.1016/j.jcmg.2019.01.044 31488253

[96] Le Ven F Freeman M Webb J Clavel MA Wheeler M Dumont É. Impact of low flow on the outcome of high risk patients undergoing transcatheter aortic valve replacement. J Am Coll Cardiol. (2013) 62:782–8. 10.1016/j.jacc.2013.05.044 23770162

[97] Dayan V Vignolo G Magne J Clavel MA Mohty D Pibarot P. Outcome and impact of aortic valve replacement in patients with preserved LV ejection fraction and low gradient aortic stenosis: a meta-analysis. J Am Coll Cardiol. (2015) 66:2594–603. 10.1016/j.jacc.2015.09.076 26670058

[98] Delgado V Bax JJ. Left ventricular stroke volume in severe aortic stenosis and preserved left ventricular ejection fraction: prognostic relevance. Eur Heart J. (2018) 39:2000–2. 10.1093/eurheartj/ehy283 29788101

[99] Bavishi C Balasundaram K Argulian E. Integration of flow-gradient patterns into clinical decision making for patients with suspected severe aortic stenosis and preserved LVEF: a systematic review of evidence and meta-analysis. J Am Coll Cardiol Img. (2016) 9:1255–63. 10.1016/j.jcmg.2016.01.035 27544900

[100] Guzzetti E Clavel MA Pibarot P. Importance of flow in risk stratification of aortic stenosis. Can J Cardiol. (2020) 36:27–9. 10.1016/j.cjca.2019.10.020 31810743

[101] Saeed S Vamvakidou A Seifert R Khattar R Li W Senior R. The impact of aortic valve replacement on survival in patients with normal flow low gradient severe aortic stenosis: a propensity-matched comparison. Eur Heart J Cardiovasc Imaging. (2019) 20:1094–101. 10.1093/ehjci/jez191 31327014

[102] Fukui M Gupta A Abdelkarim I Sharbaugh MS Althouse AD Elzomor H. Association of structural and functional cardiac changes with transcatheter aortic valve replacement outcomes in patients with aortic stenosis. JAMA Cardiol. (2019) 4:215–22. 10.1001/jamacardio.2018.4830 30725109 PMC6439547

[103] Monin JL Quere JP Monchi M Petit H Baleynaud S Chauvel C. Low-gradient aortic stenosis: operative risk stratification and predictors for long-term outcome: a multicenter study using dobutamine stress hemodynamics. Circulation. (2003) 108:319–24. 10.1161/01.CIR.0000079171.43055.46 12835219

[104] Tribouilloy C Levy F Rusinaru D Gueret P Petit-Eisenmann H Baleynaud S. Outcome after aortic valve replacement for low-flow/low-gradient aortic stenosis without contractile reserve on dobutamine stress echocardiography. J Am Coll Cardiol. (2009) 53:1865–73. 10.1016/j.jacc.2009.02.026 19442886

[105] Sato K Sankaramangalam K Kandregula K Bullen JA Kapadia SR Krishnaswamy A. Popović contemporary outcomes in low-gradient aortic stenosis patients who underwent dobutamine stress echocardiography. J Am Heart Assoc. (2019) 8:e011168. 10.1161/JAHA.118.011168 30879370 PMC6475055

[106] Ribeiro HB Lerakis S Gilard M Cavalcante JL Makkar R Herrmann HC. Transcatheter aortic valve replacement in patients with low-flow, low-gradient aortic stenosis: the TOPAS-TAVI registry. J Am Coll Cardiol. (2018) 71:1297–308. 10.1016/j.jacc.2018.01.054 29566812

[107] Hein S Arnon E Kostin S Schonburg M Elsasser A Polyakova V. Progression from compensated hypertrophy to failure in the pressure-overloaded human heart: structural deterioration and compensatory mechanisms. Circulation. (2003) 107:984–91. 10.1161/01.CIR.0000051865.66123.B7 12600911

[108] Treibel TA Kozor R Schofield R Benedetti G Fontana M Bhuva AN. Reverse myocardial remodeling following valve replacement in patients with aortic stenosis. J Am Coll Cardiol. (2018) 71:860–71. 10.1016/j.jacc.2017.12.035 29471937 PMC5821681

[109] Treibel TA López B González A Menacho K Schofield RS Ravassa S. Reappraising myocardial fibrosis in severe aortic stenosis: an invasive and non-invasive study in 133 patients. Eur Heart J. (2017) 39:699–709. 10.1093/eurheartj/ehx353 29020257 PMC5888951

[110] Musa TA Treibel TA Vassiliou VS Captur G Singh A Chin C. Myocardial scar and mortality in severe aortic stenosis. Circulation. (2018) 138:1935–47. 10.1161/CIRCULATIONAHA.117.032839 30002099 PMC6221382

[111] Tastet L Kwiecinski J Pibarot P Capoulade R Everett RJ Newby DE. Sex-related differences in the extent of myocardial fibrosis in patients with aortic valve stenosis. JACC Cardiovasc Imaging. (2020) 13:699–711. 10.1016/j.jcmg.2019.06.014 31422128

[112] Bing R Everett RJ Tuck C Semple S Lewis S Harkess R. Rationale and design of the randomized, controlled early valve replacement guided by biomarkers of left ventricular decompensation in asymptomatic patients with severe aortic stenosis (EVOLVED) trial. Am Heart J. (2019) 212:91–100. 10.1016/j.ahj.2019.02.018 30978556

[113] Lindman BR Dweck MR Lancellotti P Genereux P Pierard LA O'Gara PT. Management of asymptomatic severe aortic stenosis: evolving concepts in timing of valve replacement. JACC Cardiovasc Imaging. (2020) 13:481–93. 10.1016/j.jcmg.2019.01.036 31202751

[114] d'Arcy JL Coffey S Loudon MA Kennedy A Pearson-Stuttard J Birks J. Large-scale community echocardiographic screening reveals a major burden of undiagnosed valvular heart disease in older people: the OxVALVE population cohort study. Eur Heart J. (2016) 37:3515–22. 10.1093/eurheartj/ehw229 27354049 PMC5216199

